# Differential diversity and structure of autotrophs in agricultural soils of Qinghai Province

**DOI:** 10.1128/spectrum.02693-24

**Published:** 2025-01-08

**Authors:** Lianyu Zhou, Xuelan Ma, Qiaoyu Luo, Feng Qiao, Huichun Xie, Longrui Wang, Wenjuan Sun, Yu Liu, Yun Ma

**Affiliations:** 1 Key Laboratory of Medicinal Plant and Animal Resources of the Qinghai-Tibetan Plateau in Qinghai Province, Academy of Plateau Science and Sustainability, School of Life Science, Qinghai Normal University, Xining, China; University of Minnesota Twin Cities, St. Paul, Minnesota, USA

**Keywords:** agricultural soil, autotrophic microorganism, carbon fixation potential, community structure, RubisCO activity

## Abstract

The biodiversity of CO_2_-assimilating bacterial communities is pivotal for carbon sequestration in agricultural systems. Changes in the diversity, structure, and activity of the soil chemolithoautotrophic bacteria were examined in four agricultural areas, Dulan (DL), Gonghe (GH), Huzhu (HZ), and Datong (DT) counties in Qinghai Province, where wheat, oilseed rape, and barley were planted. This process was performed using Illumina amplicon sequencing of the ribulose-1,5-bisphosphatecarboxylase/oxygenase (RubisCO) gene (*cbbL* Form I) and activity data. The diversity, community, and activity of soil autotrophic CO_2_-fixing bacteria differed significantly across soil sites, whereas *cbbL*-bearing bacterial diversity and activity were similar across different crop types. RubisCO activity in the HZ region was significantly greater than in the other three regions (*P* < 0.001). The overall relative abundance trend of the bacterial taxa was similar among the three crop samples. Moreover, 31, 27, 10, and 8 significant linear discriminant analysis effect sizes were identified in the four regions collected from HZ, DL, DT, and GH, respectively. No significant biomarkers were detected in any of the crop groups. Some soil properties had significant relationships with the autotrophic bacterial community composition.

**IMPORTANCE:**

Agricultural soil plays important roles in carbon fixation during carbon capture and storage. Autotrophic bacteria that utilize inorganic compounds as electron donors for growth fix CO_2_ photosynthetically or chemo-autotrophically in diverse ecosystems and affect soil organic carbon sequestration. Soil properties, agronomic management measures, and environmental factors can influence the community composition, abundance, and activity of CO_2_-assimilating bacteria. This study aims at evaluating the effects of different regions and crop types on the abundance, composition, and activity of CO_2_-fixing bacteria in agricultural soil.

## INTRODUCTION

The concentration of carbon dioxide in the atmosphere is continuously increasing, posing a threat to global warming. Atmospheric CO_2_ is primarily removed by biological CO_2_ fixation by terrestrial plants or microbes (1, 2). Biological systems for CO_2_ conversion provide a potential path forward owing to their high application selectivity and adaptability. Moreover, many bacteria can use CO_2_ as their sole source of carbon and convert it into value-added products (3). Autotrophic bacteria that utilize inorganic compounds as electron donors for growth fix CO_2_ photosynthetically or chemo-autotrophically even in extreme ecosystems, and affect soil organic carbon sequestration. Among the seven pathways developed by autotrophic bacteria for carbon dioxide fixation, the Calvin-Benson-Bassham (CBB) cycle is the most widely distributed pathway (4–9). Ribulose-1,5-bisphosphate carboxylase oxygenase (RubisCO) is the rate-limiting enzyme of the CBB pathway and exists in forms I, II, III, and IV, each with different structures, catalytic properties, and substrate specificities. Among the four forms, form I RubisCO is the most abundant in autotrophic bacteria (10–12). The *cbbL* gene, which encodes a large subunit of RubisCO form I, has been widely used as a functional marker for studying autotrophic CO_2_-fixing bacteria in diverse ecosystems (13–24).

Agricultural soils have drawn increasing attention as important components of terrestrial ecosystems for carbon fixation during carbon capture and storage with long-term effects on ecosystems. Soil microbial fixation of atmospheric CO_2_ notably contributes to the organic C pool in agricultural soils under various management conditions (25, 26). Previous studies that have focused on *cbbL*-containing bacteria have demonstrated that soil properties, agronomic management measures, and environmental factors can influence the community composition, abundance, and activity, although the extent to which each factor plays a role has varied among these studies (14, 25, 27–34). For example, the predominance of *cbbL* sequences is associated with *Rhizobium leguminosarum*, *Bradyrhizobium* sp., *Sinorhizobium meliloti*, *Ochrobactrum anthropi*, and a variety of uncultured *cbbL*-harboring bacteria from the rhizosphere of *Arachis hypogaea* (27). Additionally, *cbbL*-containing bacterial communities and diversity indices vary with site and plant type in agroecosystem soils (14, 35).

In Qinghai Province, agricultural soils cover an area of approximately 58.97 million ha. Wheat, barley, and oilseed rape are the main crops grown in semiarid regions. Previously, differences in the diversity of the 16S rRNA and ITS1 genes were observed in the rhizosphere soils of oats in different alpine regions (36). The role of soil microbial autotrophy in the sequestration of carbon dioxide through the CBB pathway in both upland and paddy soils in South China has been highlighted in several studies (37–41). However, little is known about the abundance, composition, and activity of autotrophic CO_2_ fixation microorganisms in the semiarid alpine region of northwestern China.

In the present study, four areas of soil were directly collected from a field in Qinghai Province, China, where wheat, oilseed rape, and barley were planted as major crops once each year. Field sampling was conducted during the flowering and fruiting stages. Autotrophic CO_2_-assimilating bacterial diversity and communities were assessed by *cbbL* gene amplicon sequencing. This study aimed to determine the effects of different regions and crop types on the abundance, composition, and activity of CO_2_-fixing bacteria in agricultural soil.

## MATERIALS AND METHODS

### Field sampling

Field sampling in Qinghai Province (36°02′18.36″–37°04′10.24″N, 98°03′40.64″–102°06′08.23″E) was conducted as previously described by Zhou et al. (42). Briefly, on July 2022, 9, 4, 12, and 15 soil samples were collected from Dulan (DL), Datong (DT), Gonghe (GH), and Huzhu (HZ) counties (Fig. S1; Table S1), respectively. The mean annual precipitation in DL county is 179.1 mm, with a mean annual temperature ranging from 16°C to 27°C. DT county experiences an average annual precipitation of 450–800 mm, with a low temperature of 10°C and a high temperature of 24°C. The GH region has an average high temperature of 23°C, a low temperature of 10°C, and receives between 250 and 420 mm of precipitation. In the HZ region, the mean annual temperature ranges from 16°C to 27°C, with precipitation levels varying from 350 to 650 mm. The cropping system was wheat (*Triticum aestivum*, Ta), oilseed rape (*Brassica napus*, Bn), and barley (*Hordeum vulgare*, Hv), respectively. Considering that environmental factors affect the crop planting area, sites in the same village were chosen to minimize the effects of these factors. Barley was planted narrowly in DT County. Each sample was collected from 5 to 20 cm surface soil using a drill (10 cm in diameter) by mixing five soil cores. The samples for each plot were divided into three parts and stored at 4°C, –20°C, and –80°C for the measurement of soil biogeochemical properties, soil enzymes, and soil DNA extraction.

### Microbial DNA extraction and Illumina high-throughput sequencing

Microbial DNA was extracted from the soil samples using a HiPure soil DNA kit (Azenta) according to the manufacturer’s protocol. The DNA concentration was monitored using the Qubit dsDNA HS Assay Kit. A sequencing library was constructed using a MetaVX library preparation kit (Azenta, Inc., South Plainfield, NJ, USA). The *cbbL* gene was amplified with primers 595f (5′-GACTTCACCAAAGACGACGA-3′) and 1387r (5′-TCGAACTTGATTTCTTTCCA-3′) (43). PCR was conducted using the following program: 3 min of denaturation at 94°C, 24 cycles of 5 s at 95°C, 90 s of annealing at 57°C, and 10 s of elongation at 72°C, and a final extension at 72°C for 5 min. Another 25 µL of PCR mixture was prepared with 2.5 µL of TransStart buffer, 2 µL of dNTPs, 1 µL of each primer, 0.5 µL of TransStart Taq DNA polymerase, and 20 ng of template DNA. Indexed adapters were added to each amplicon using a limited-cycle PCR. Subsequently, magnetic beads were used to purify the library.

### Illumina sequencing and data processing

The concentration of PCR product was determined using a microplate reader (Tecan, Infinite 200 Pro), and the amplicon size was checked using agarose gel (1.5%) electrophoresis. Next-generation sequencing was analyzed on an Illumina MiSeq platform (Illumina, San Diego, USA) at Azenta, Inc. (South Plainfield, NJ, USA). Automated cluster generation and 250/300 base paired-end sequencing with dual reads were performed following the manufacturer’s protocol.

The raw sequences were extracted, trimmed, and quality screened using Quantitative Insights into Microbial Ecology (QIIME, version 1.9.1). Briefly, quality filtering of the joined sequences was performed, and low-quality sequences (quality score <20, length <200 bp) were discarded. After quality filtering, the sequence data set was used for taxonomic grouping, and unique sequences were aligned to the SILVA 138 database with a 70% confidence threshold. The remaining sequences were binned into operational taxonomic units (OTUs) with a 97% nucleotide sequence similarity cutoff using Vsearch (version 1.9.6). The Bayes algorithm classifier provided by the Ribosomal Database Program (RDP, version 2.2) was used to analyze the representative sequences of OTUs.

Based on the OTU analysis results, the Good’s coverage and Alpha-diversity (Shannon, Simpson, Chao1, and Ace) of the autotrophic bacterial communities were calculated using QIIME. In addition, the relative abundances of dominant taxa including the phylum, class, order, family, and genus, were estimated by the proportion of number of reads allocated to a particular taxon to the total obtained sequences. The Venn diagram and the relative abundance of dominant taxa were determined using R software (version 3.3.1). Nonmetric multidimensional scaling (NMDS) was performed using the vegan R package with normalized data. Analysis of similarity (ANOSIM) tests were also conducted to assess the roles of region and crop type in structuring CO_2_-assimilating bacterial communities. The R package was used to cluster samples based on the Bray-Curtis similarity index by using principal coordinates analysis (PCoA). Linear discriminant analysis effect size (LEfSe, version 1.0) was applied to identify the biomarker taxa that explained the differences between different groups using linear discriminant analyses (LDAs = 2).

### Soil physical and chemical analysis

Total protein was extracted from the soil according to the instructions of the RubisCO assay kit (Jiangsu Meimian Industrial Company, Jiangsu, China). Briefly, 1 g of soil was suspended in 9 mL of 0.01 mol/L phosphate-buffered saline (pH 7.2). The mixture was manually homogenized in a mortar and pestle. The extract was centrifuged at 4°C for 15 min at 5,000 rpm. RubisCO activity was monitored at 450 nm by using a BioStach Ready (BioTek Instruments, Inc., Winooski, USA). Reactions in the absence of soil extraction and enzyme-labeled reagents were used as negative controls. Other physical and chemical properties, including pH, soil water content, organic matter, ammonium nitrogen, nitrate nitrogen, total phosphate, effective phosphate, total sulfur, and effective sulfur, were reported in a previous study (42).

### Statistical analysis

The data are expressed as the mean ± standard deviation. A univariate analysis of variance (ANOVA) was performed to determine between-subject effects in accordance with the general linear model. Correlation analysis was performed between the measured indicators and environmental factors using Pearson test (SPSS 16).

## RESULTS

### α-diversity of the autotrophic microbial community

**Fig 1:**
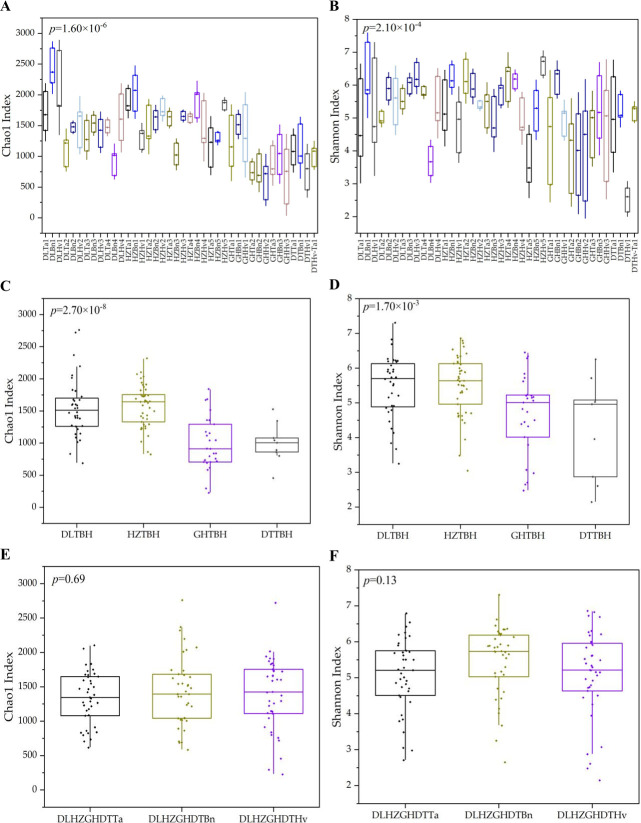
Chao1 and Shannon diversity indices in 40 soil samples (**A, B**), four region groups (**C, D**) and three crop types (**E, F**). The abscissa is the name of the soil sample. The boxplots represent medians and each sample. DLTa, HZTa, GHTa, and DTTa represent wheat soils from DL, HZ, GH, and DT regions, respectively. DLBn, HZBn, GHBn, and DTBn represent oilseed rape soils from DL, HZ, GH, and DT regions, respectively. DLHv, HZHv, GHHv, and DTHv represent barley soils from DL, HZ, GH, and DT regions, respectively. 1, 2, 3, 4, or 5 represent the number of soil samples. DLHvDLTBH: DL region soils; HZTBH: HZ region soils; GHTBH: GH region soils; DTTBH: DT region soils; DLHZGHDTTa: wheat soils; DLHZGHDTBn: oilseed rape soils; and DLHZGHDTHv: barley soils.

The Chao1 and Ace indices, which are the two key estimators for calculating the community richness, ranged from 616.63 ± 286.44 to 2,442.06 ± 289.16 and from 602.98 ± 279.67 to 2,523.04 ± 264.80, respectively (Fig. 1A and B; Tables S2 to S5). The Simpson and Shannon diversity indices ranged from 0.551 ± 0.040 to 0.962 ± 0.007 and from 2.54 ± 0.37 to 6.63 ± 0.29, respectively. High values of the Chao1 and Ace indices were observed for DLBn1, whereas low values were obtained for GHHv2. In contrast, high Simpson and Shannon diversity indexes were observed for HZHv5, whereas low values were observed for DTHv1. The Chao1 and Shannon diversity indices indicated that the diversity of the *cbbL* gene differed significantly among the 40 soil samples (*P* < 0.001).

Regions with highly significant (*P* < 0.001, Table 1) effects were detected for the Ace, Chao1, Shannon, Simpson, and Goods coverage indices of the *cbbL* gene. Crop type had the most significant (*P* < 0.01) effect on the *cbbL* gene according to Simpson and Shannon indices. In addition, Simpson and Shannon diversity indices exhibited interactions between region and crop type (*P* < 0.05, Table 1). Among the four regions, the Chao1 and Shannon diversity indices in the DL and HZ samples were significantly greater than those in the GH and DT soils (*P* < 0.05, Fig. 1C and D). According to the analysis of the effect of crop type on community composition using one-way ANOVA, no significant differences were observed in the Chao1 or Shannon indices across the three crop groups (Fig. 1E and F). The Chao1 and Shannon indices significantly varied in the each region across different crops, as well as in the planted crop across various regions (Fig. S2; Tables S2 to S5). Except for the DT location, there were no significant differences in α-diversity indexes between barley and wheat soils at the same site. However, in certain sampling location, the Chao1 and Shannon diversity indices varied significantly between oilseed rape and other crop soils.

**TABLE 1:** Results of the univariate analysis for the effects of region and crop on autotrophic bacterial α-diversity indexes, genera, and RubisCO activity

Bacterial indexes or RubisCO activity	Region		Crop		Region × Crop	
	*F*	*p*	*F*	*p*	*F*	*p*
Ace	18.080	0.000	0.744	0.478	0.808	0.566
Chao1	17.919	0.000	0.727	0.486	0.767	0.598
Shannon	8.135	0.000	5.248	0.007	2.751	0.016
Simpson	6.637	0.000	10.183	0.000	5.090	0.000
Goods coverage	17.765	0.000	0.704	0.497	0.734	0.623
RubisCO activity	5.188	0.002	0.004	0.996	0.832	0.548
*Pseudonocardia*	2.681	0.051	2.517	0.086	1.470	0.195
*Sulfuritortus*	32.653	0.000	0.872	0.421	2.904	0.012
Unclassified genus	2.60	0.053	0.418	0.660	1.567	0.164
*Sulfuricaulis*	12.453	0.000	0.115	0.892	1.246	0.289
*Hydrogenophaga*	15.031	0.000	0.059	0.943	0.683	0.663
*Nitrobacter*	4.860	0.003	0.441	0.644	3.114	0.008
*Nitrosomonas*	8.443	0.000	0.036	0.964	0.039	0.993
*Thiohalobacter*	4.275	0.007	2.777	0.067	2.972	0.010
*Beggiatoa*	3.408	0.020	1.337	0.267	2.243	0.045
*Limnohabitans*	2.208	0.092	0.133	0.876	1.021	0.416

### β-diversity of autotrophic microbial community

**Fig 2:**
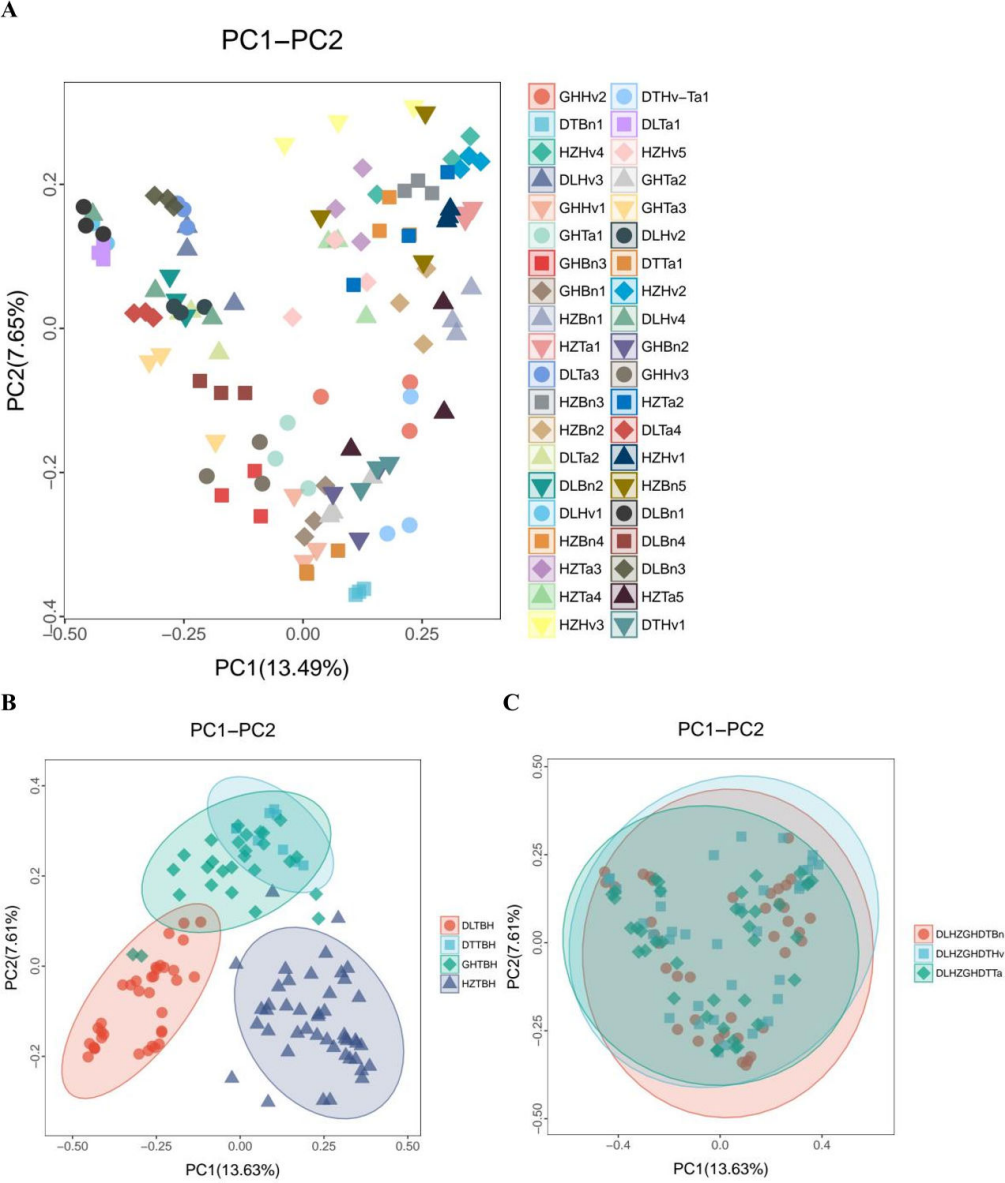
Plots of principal coordinate analysis (PCoA) of the microbial community structure of 40 different soils (**A**), four region groups (**B**), and three crop types (**C**). Each point represents a sample. Samples from the same group are marked as the same color.

PCoA based on the Bray-Curtis dissimilarity revealed that the *cbbL*-containing bacterial community extracted from the soil samples differed (Fig. 2A). The PCoA indicated that the bacterial communities from the GH, DT, and DL regions were more similar than those from the HZ region (Fig. 2B). However, the results for oilseed rape, wheat, and barley soils mostly overlapped (Fig. 2C).

**Fig 3:**
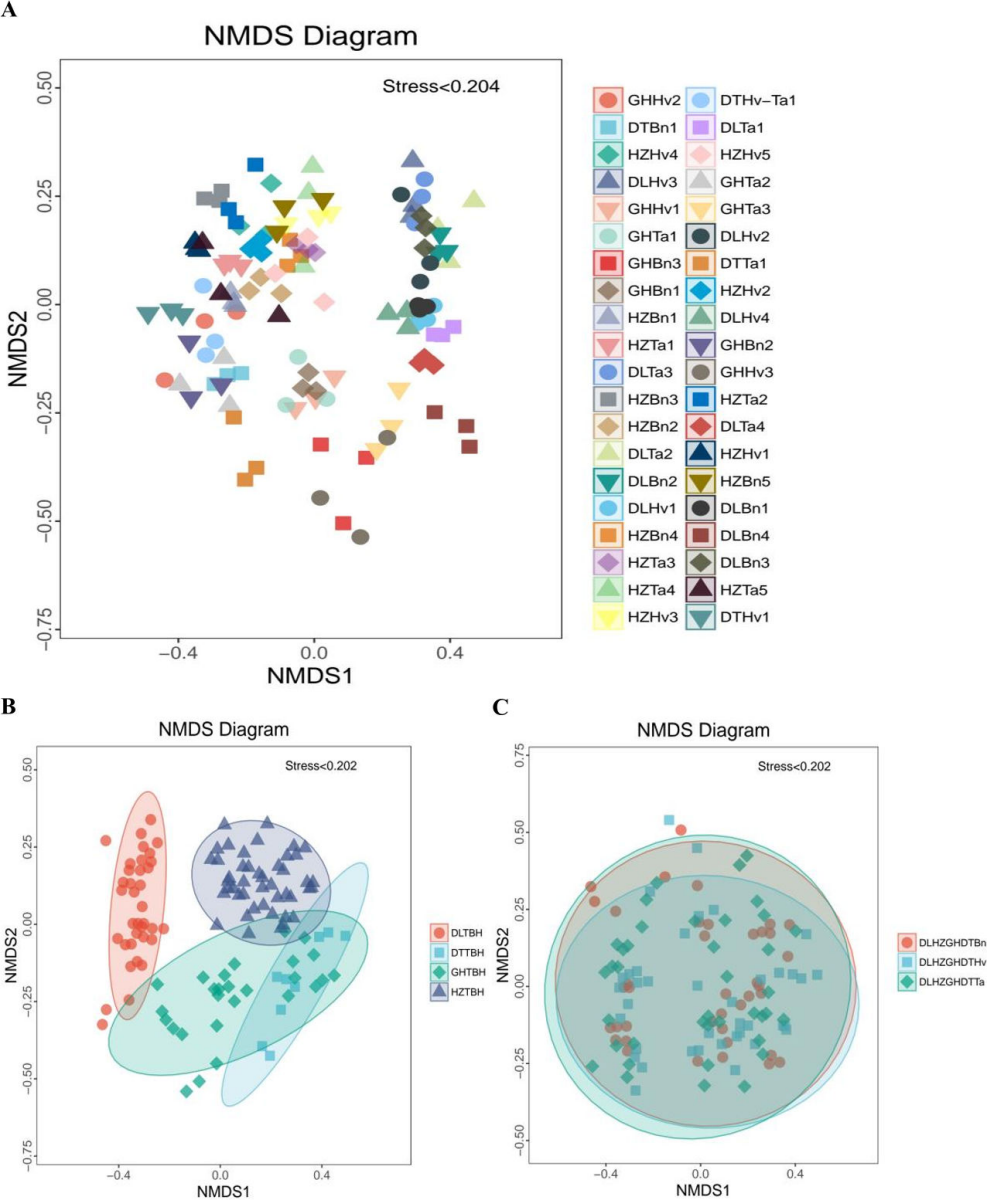
Nonmetric multidimensional scaling (NMDS) ordination pattern of the *cbbL*-containing bacterial community composition in 40 soil samples (**A**), four region groups (**B**), and three crop types (**C**). Each point represents a sample, and the distance between points represents the degree of variation. Samples from the same group are marked as the same color.

The distinct distributions of the *cbbL*-containing bacterial communities were compared among the 40 soil samples using NMDS analysis (Fig. 3A). The HZ, DL, GH, and DT regions were largely separated (Fig. 3B, ANOSIM, *R* = 0.75, *P* = 0.001), revealing a clear distinction in the *cbbL*-containing bacterial communities by region. Within all three crop groups, the NMDS plot ordination estimated the distribution of *cbbL*-containing bacterial communities between and within clusters (Fig. 3C, *R* = –0.008, *P* = 0.667). However, NMDS ordination plotting revealed a highly significant separation in each region across different crops, as well as in the planted crop across different regions (Fig. S3 and S4).

### Autotrophic microbial OTUs

**Fig 4:**
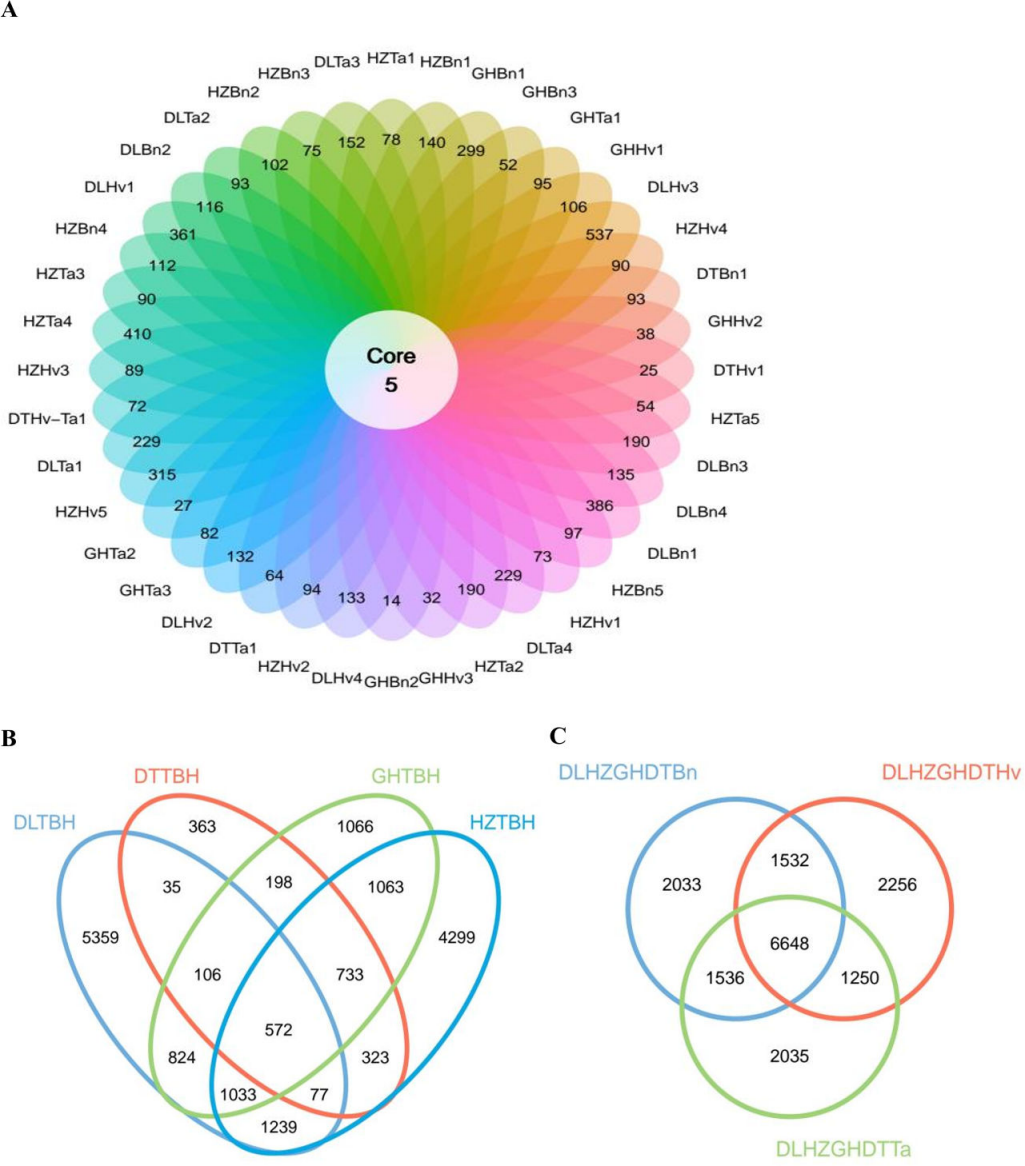
Number of shared and unique OTUs between 40 soil samples (**A**), four region groups (**B**), and three crop types (**C**). DLTBH, HZTBH, GHTBH, and DTTBH represent the DL, HZ, GH, and DT soils, respectively. DLHZZGHDTTa, DLHZZGHDTBn, and DLHZZGHDTHv represent the wheat, oilseed rape, and barley soils, respectively.

Forty samples collected from these four regions were successfully amplified and sequenced. *cbbL* clone sequences were grouped into OTUs based on a cutoff of 97% sequence similarity. The number of OTUs ranged from 31,068 to 84,984 per soil sample. The average number of OTUs was 55,178.058, and the highest number was observed for DLTa1. A total of 17,290 OTUs were identified, 5 of which (OTU1, OTU2, OTU5, OTU36, and OTU26) were among the 40 soil groups (Fig. 4A). A total of 537, 410, 386, 361, and 315 OTUs were unique to DLHv3, HZTa4, DLBn1, DLHv1, and HZHv5, respectively.

In total, 9,339, 9,245, 5,595, and 2,407 OTUs were obtained from the HZ, DL, GH, and DT soils, respectively (Fig. 4B). Among the different regions, a large overlap of bacterial OTUs was observed in the GH and HZ soils, whereas the DL, GH, HZ, and DT soils shared 572 OTUs. Furthermore, only 5,359 OTUs were present in the DL soils, 4,299 OTUs were present in the HZ soils, 1,066 OTUs were present in the GH soils, and 363 OTUs were present in the DT soils.

Moreover, 11,749, 11,686, and 11,469 OTUs were obtained from the oilseed rape, barley, and wheat soils, respectively (Fig. 4C). Among the different crops, the three crops shared 6,648 common OTUs, only 2,033 OTUs were detected in oilseed rape soils, 2,035 OTUs were found in wheat soils, and 2,256 OTUs were observed in barley soils.

### Autotrophic microbial community composition

**Fig 5:**
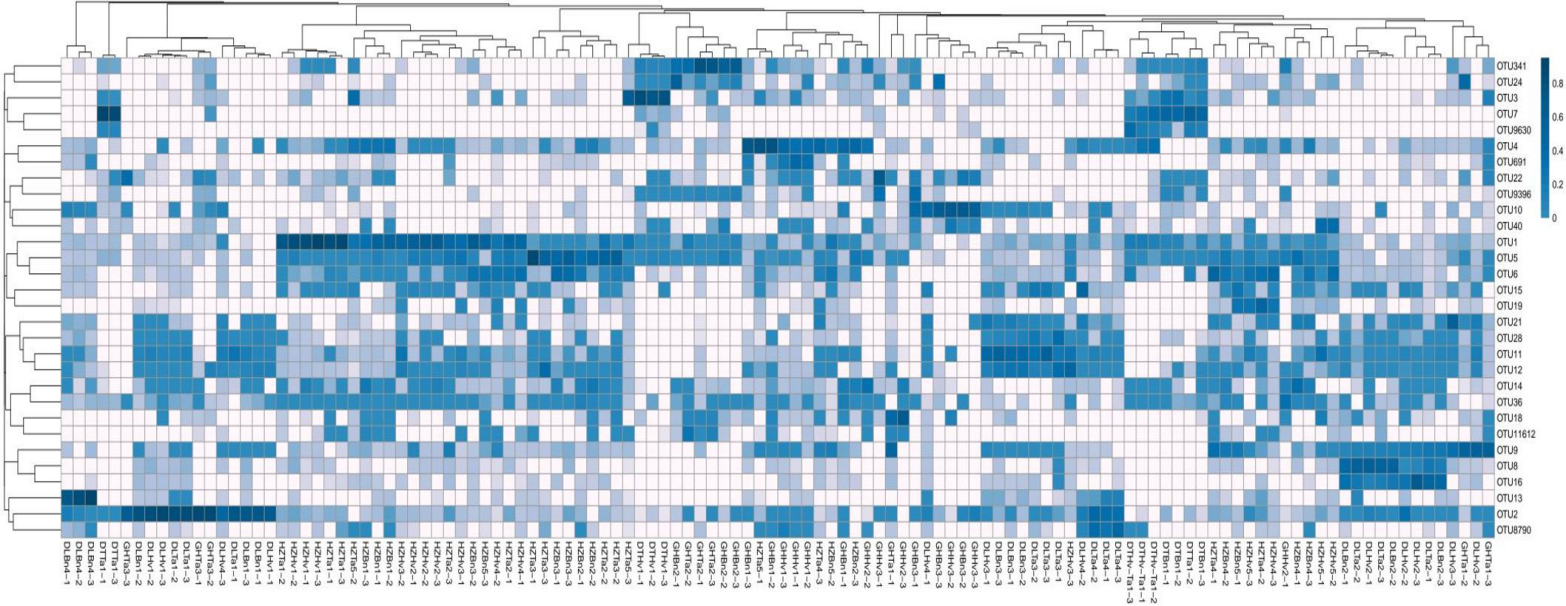
Heatmap of the 30 highly abundant OTUs for community differences. The abscissa is the name of the soil sample and sample repetitions. Each row represents a different OTU.

Figure 5 shows a distinct heatmap of the 30 selected dominant OTUs across all the samples. The coverage percentage of the top 30 OTUs varied among the samples. The coverage rates of OTU36, OTU14, OTU12, and OTU6 in the soil samples were 97.5%, 95%, 92.5%, and 90%, respectively. OTU7 and OTU10 had low coverage (25% and 17.5%, respectively). OTU1 was primarily distributed in the HZTa1 and HZHv1 soils. OTU2 was dominant in the DLTa1, DLBn1, and DLHv1 soils. The highest number of OTU3 or OTU7 was observed in the DTHv1 or DTTa1 soils. OTU5 was primarily distributed in the HZTa3 soil, whereas OTU13 was primarily present in the DLBn4 soil. In addition, OTU341 dominated the GHTa2 and GHBn2 soils.

**Fig 6:**
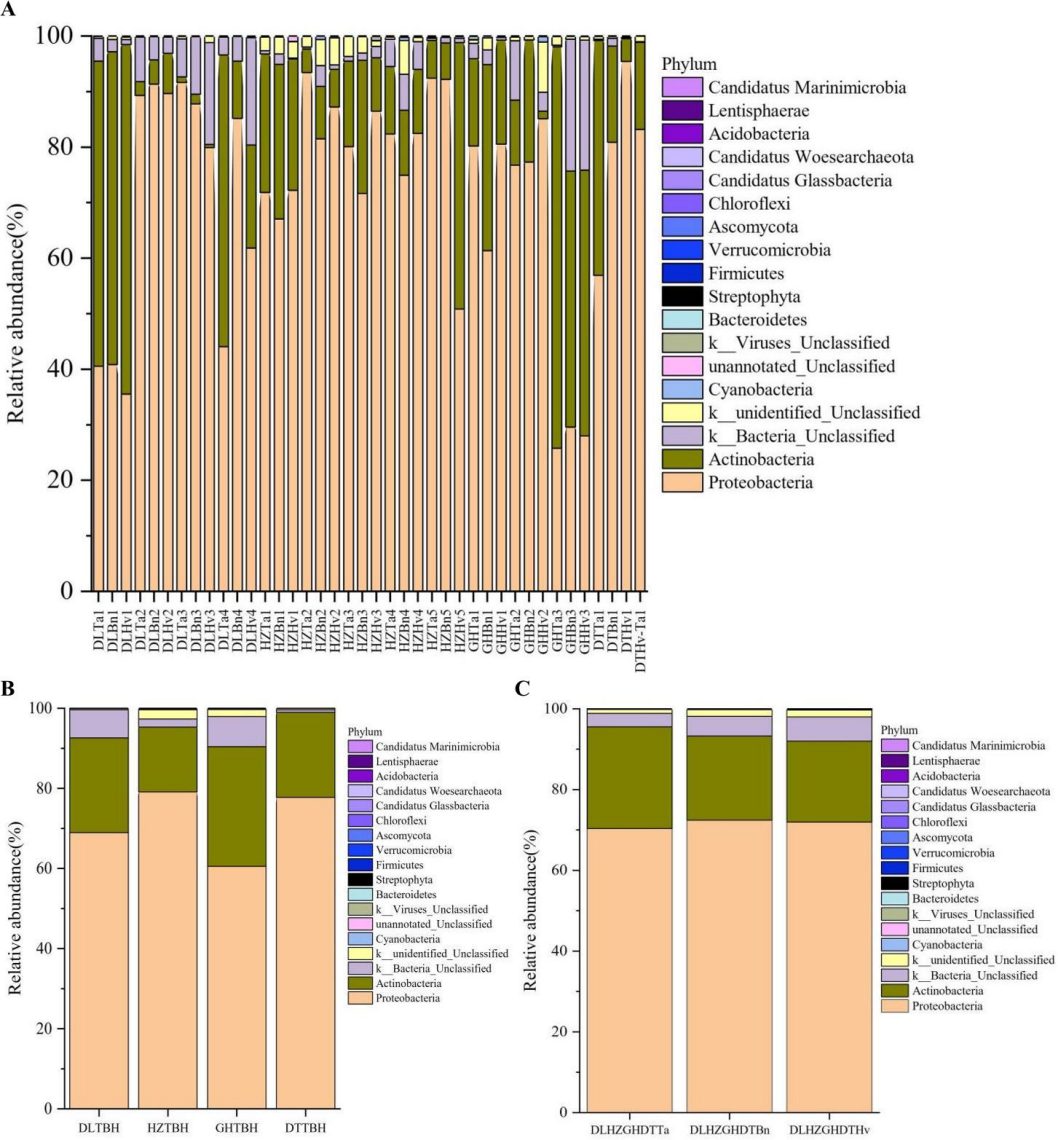
Distribution and relative abundances of the dominant phyla in 40 soil samples (**A**), four region groups (**B**), and three crop types (**C**). The abscissa is the name of the soil sample. The ordinate is the relative abundance of different phyla. The right color in the bar chart corresponds to the phylum name of the following legend.

The number of taxa at the phylum, class, order, family, and genus levels was calculated. In total, 18 phyla were identified (Fig. 6A). Proteobacteria and Actinobacteria were detected across all the samples and composed the top two phyla, with relative abundances ranging from 50.87% to 95.44% and from 46.08% to 74.68%, respectively. Differences were observed in the phylum composition within the regional groups (Fig. 6B). Among the top two phyla, Proteobacteria and Actinobacteria accounted for 71.02% and 32.50%, respectively, of the bacteria in the DL group; 79.11% and 16.17%, respectively, in the HZ group; 60.52% and 29.91%, respectively, in the GH group; and 77.75% and 21.22%, respectively, in the DT group, In addition, the abundance of Proteobacteria in the three crop types was similar (Fig. 6C), but the abundance of Actinobacteria decreased in the order wheat (33.35%) > oilseed rape (20.85%) > barley (20.06%).

**Fig 7:**
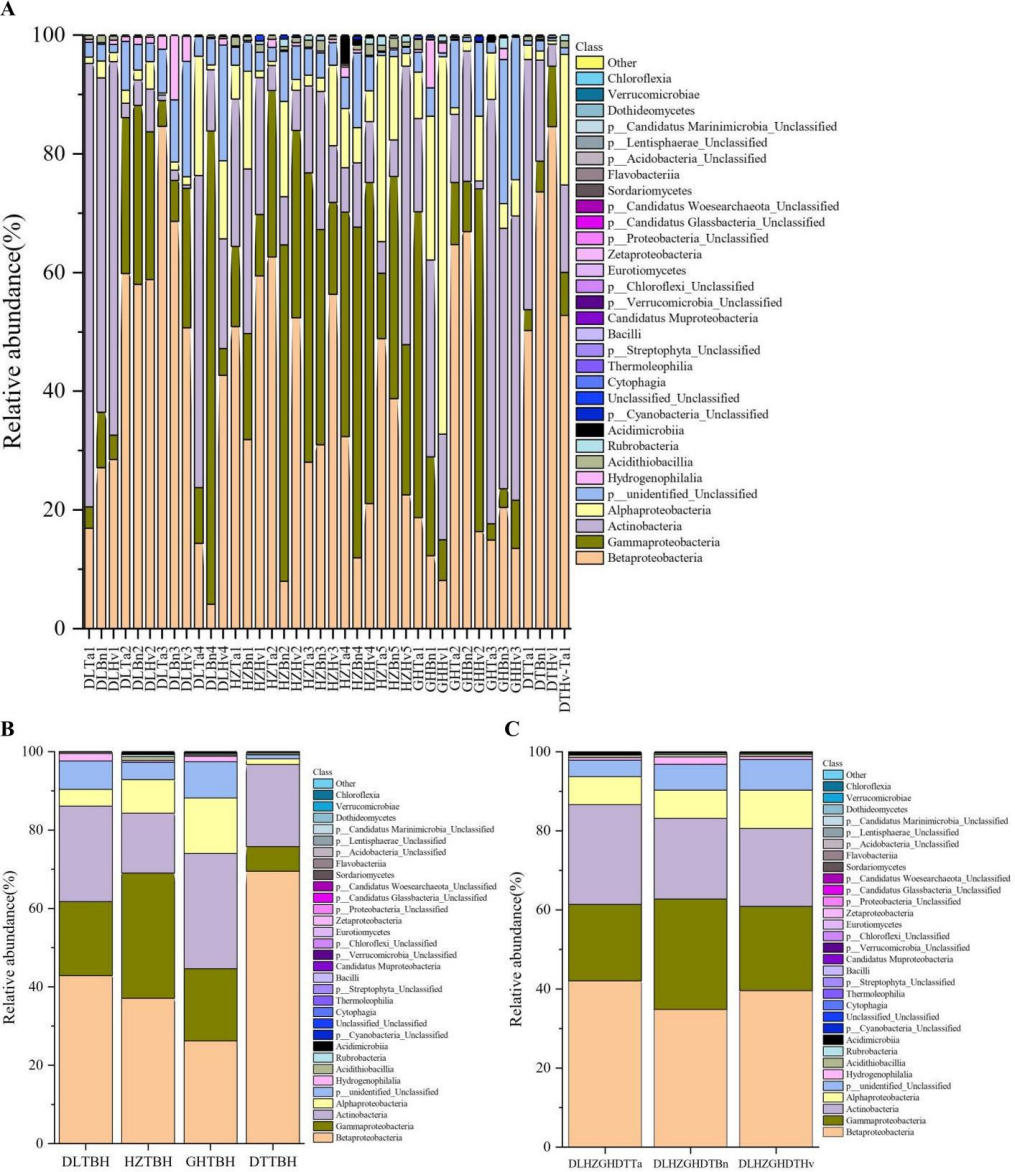
Distribution and relative abundances of the dominant classes in 40 soil samples (**A**), four region groups (**B**), and three crop types (**C**). The abscissa is the name of the soil sample. The ordinate is the relative abundance of different classes. The right color in the bar chart corresponds to the class name of the following legend. Other represents the taxonomic name except for the 30 classes of relative abundance.

A total of 32 classes were identified in this study. A diverse community was observed in each soil sample at the class level (Fig. 7A). Betaproteobacteria, Gammaproteobacteria, Actinobacteria, Alphaproteobacteria, and Acidithiobacillus were ubiquitous in the soils investigated. The soil indicator classes included mainly Betaproteobacteria, Gammaproteobacteria, Actinobacteria, and Alphaproteobacteria, with relative abundances of 31.85%–84.60%, 31.61%–79.76%, 33.16%–74.68%, and 63.61%, respectively. Notably, variations at the class level were observed in the DL, GH, HZ, and DT regions (Fig. 7B). The soil in the DL region was dominated by Betaproteobacteria (42.85%), Gammaproteobacteria (18.91%), and Actinobacteria (24.36%), whereas the GH soil was dominated by Betaproteobacteria (26.21%), Gammaproteobacteria (18.42%), Actinobacteria (29.41%), and unclassified classes (14.14%). In the HZ soils, Betaproteobacteria (37.06%), Gammaproteobacteria (32.00%), and Actinobacteria (15.27%) were the three major classes, and in the DT soils, Betaproteobacteria (69.47%) and Actinobacteria (20.96%) were the predominant classes. The three crop types had similar class compositions (Fig. 7C). Betaproteobacteria dominated 34.81%–42.08% of the bacteria in both samples, followed by Gammaproteobacteria (19.31%–27.97%) and Actinobacteria (19.72%–25.22%).

**Fig 8:**
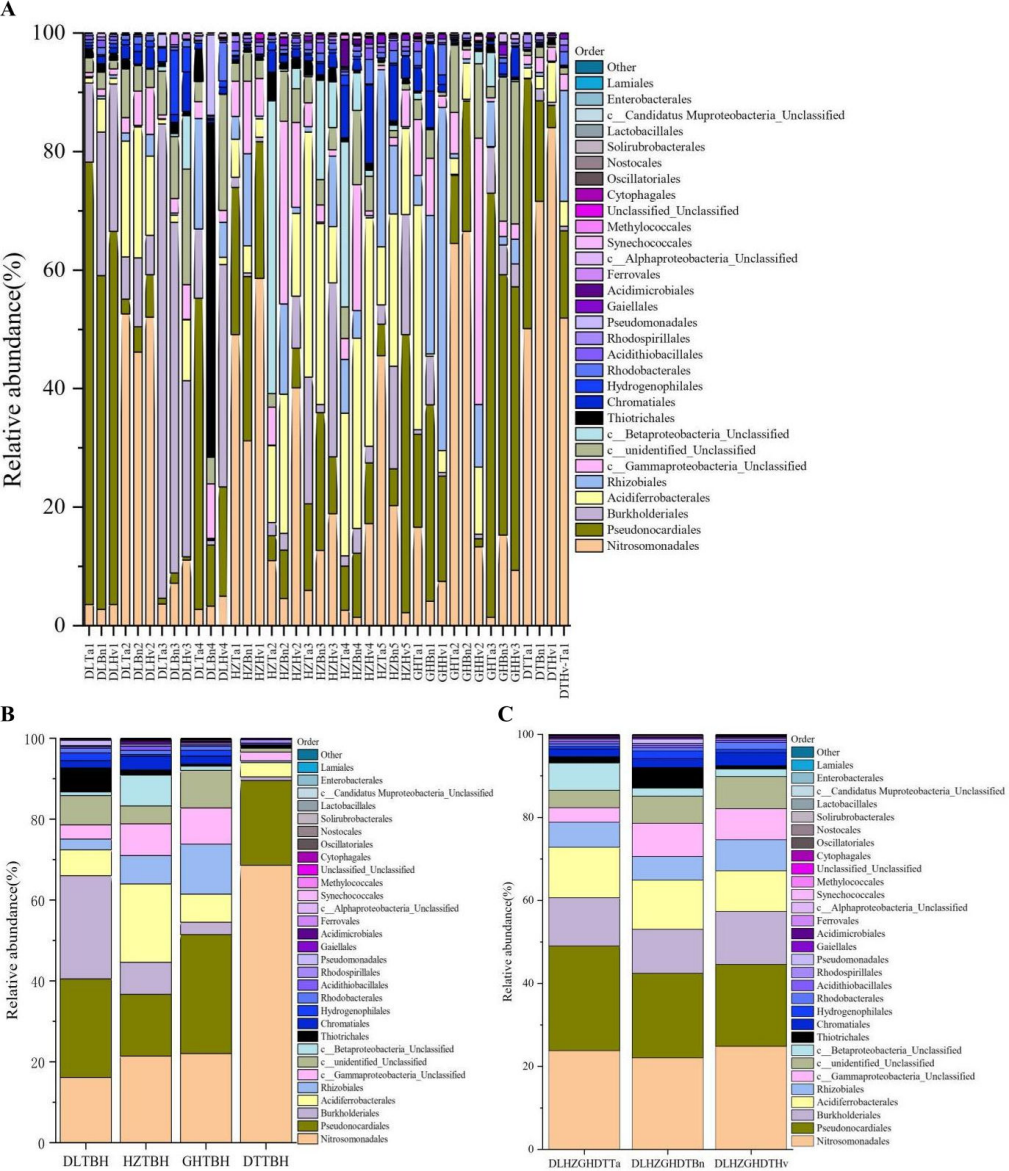
Distribution and relative abundances of the dominant orders in 40 soil samples (**A**), four region groups (**B**), and three crop types (**C**). The abscissa is the name of the soil sample. The ordinate is the relative abundance of different orders. The right color in the bar chart corresponds to the order name of the following legend. Other presents the taxonomic name except for the 30 orders of relative abundance.

A total of 47 bacterial orders were found, 11 of which were common to the tested soil samples (Fig. 8A). Nitrosomonadales (31.17%–84.07%), Pseudonocardiale (33.16%–74.68%), Burkholderiales (29.37%–80.04%), and Acidiferrobacterales (25.74%–41.31%) were highly abundant in some soils. Similarly, the GHHv1 soil was dominated by Rhizobiales (57.90%), and the DLBn4 soil was dominated by Thiotrichales (56.44%). A large fraction of these keystone orders were unclassified bacteria related to Gammaproteobacteria in the HZBn2 (30.82%) and GHHv2 (44.97%) soils, while the most abundant were unclassified bacteria associated with Betaproteobacteria in the HZTa4 (27.91%) and HZTa2 (49.39%) soils. In terms of the four regions, diverse order compositions were observed (Fig. 8B). The three orders Nitrosomonadales, Pseudonocardiales, and Burkholderiales were dominant, with relative abundances of 16.13%, 24.36%, and 25.56%, respectively, in the DL soils. Additionally, Nitrosomonadales (22.06%), Pseudonocardiales (29.41%), and Rhizobiales (12.38%) were dominant in the GH soils, and Nitrosomonadales (21.43%), Pseudonocardiales (15.27%), and Acidiferrobacterales (19.37%) were highly abundant in the HZ soils. The DT soil was dominated mainly by Nitrosomonadales (68.59%) and Pseudonocardiales (20.96%). In contrast, the orders Nitrosomonadales, Pseudonocardiales, Burkholderiales, and Acidiferrobacterales were dominant in all three crop soils (Fig. 8C). The relative abundance of Pseudonocardiales was highest in wheat soils and lowest in barley soils (25.22% and 19.72%, respectively), whereas the abundance of Nitrosomonadales was highest in wheat soils and lowest in oilseed rape soils (23.80% and 22.08%, respectively). These changes indicated that the effect of region on the order community was more evident than on the crop groups.

**Fig 9:**
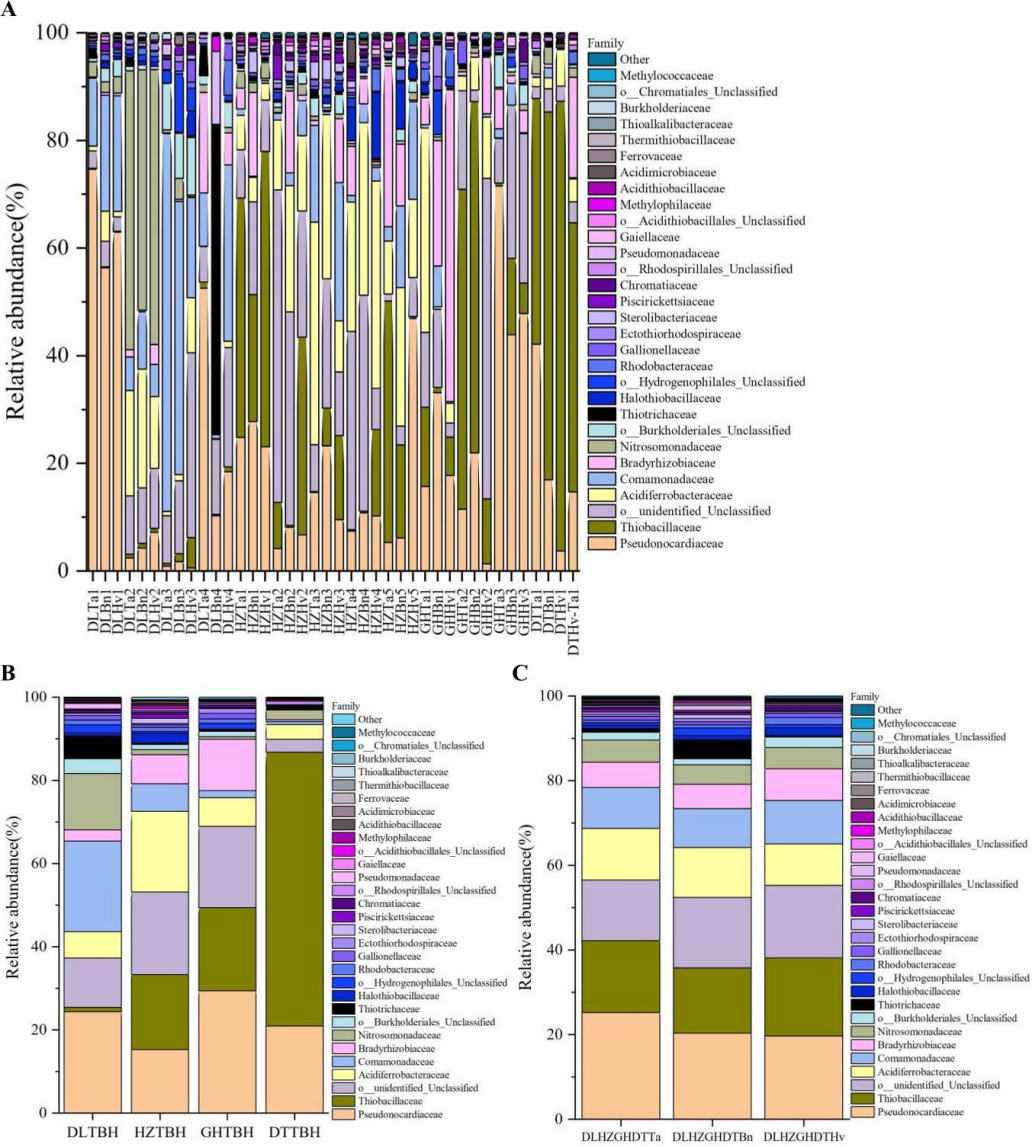
Distribution and relative abundances of the dominant families in 40 soil samples (**A**), four region groups (**B**), and three crop types (**C**). The abscissa is the name of the soil sample. The ordinate is the relative abundance of different families. The right color in the bar chart corresponds to the family name of the following legend. Other presents the taxonomic name except for the 30 families of relative abundance.

A total of 78 families were identified, and 11 common families were recorded among the 40 soils (Fig. 9A). Members of Pseudonocardiaceae (27.75%–74.68%), Thiobacillaceae (36.72%–83.56%), Acidiferrobacteraceae (25.74%–41.31%), Comamonadaceae (25.71%–70.22%), Bradyrhizobiaceae (51.78%–57.90%), Nitrosomonadaceae (44.61%–51.05%), Thiotrichaceae (56.37%), and unclassified family (34.37%–59.57%) were dominant in the 10, 10, 5, 4, 2, 2, 1, and 6 soil samples, respectively. Large differences were apparent in the taxonomic distribution of CO_2_-fixing bacteria in the four regions at the family level (Fig. 9B). The four predominant families in the DL soil were Pseudonocardiaceae (24.36%), Comamonadaceae (21.80%), Nitrosomonadaceae (13.55%), and unclassified bacteria (11.87%). The four families Pseudonocardiaceae (29.41%), Thiobacillaceae (19.99%), unclassified family (19.52%), and Bradyrhizobiaceae (12.35%) were dominant in the GH soils. The major families in the HZ soils were unclassified bacteria (19.87%), Acidiferrobacteraceae (19.37%), Thiobacillaceae (18.03%), and Pseudonocardiaceae (15.27%). Thiobacillaceae (65.82%) and Pseudonocardiaceae (20.96%) were predominant in the DT soils. In addition, the five dominant families, Pseudonocardiaceae, Thiobacillaceae, unclassified bacteria, Acidiferrobacteraceae, and Comamonadaceae accounted for abundances of 19.72%–25.22%, 15.39%–18.42%, 14.33%–17.14%, 9.79%–12.18%, and 9.15%–10.27%, respectively, of the bacteria in all three crop soils (Fig. 9C). Thus, region had a stronger effect on these families than crop type.

**Fig 10:**
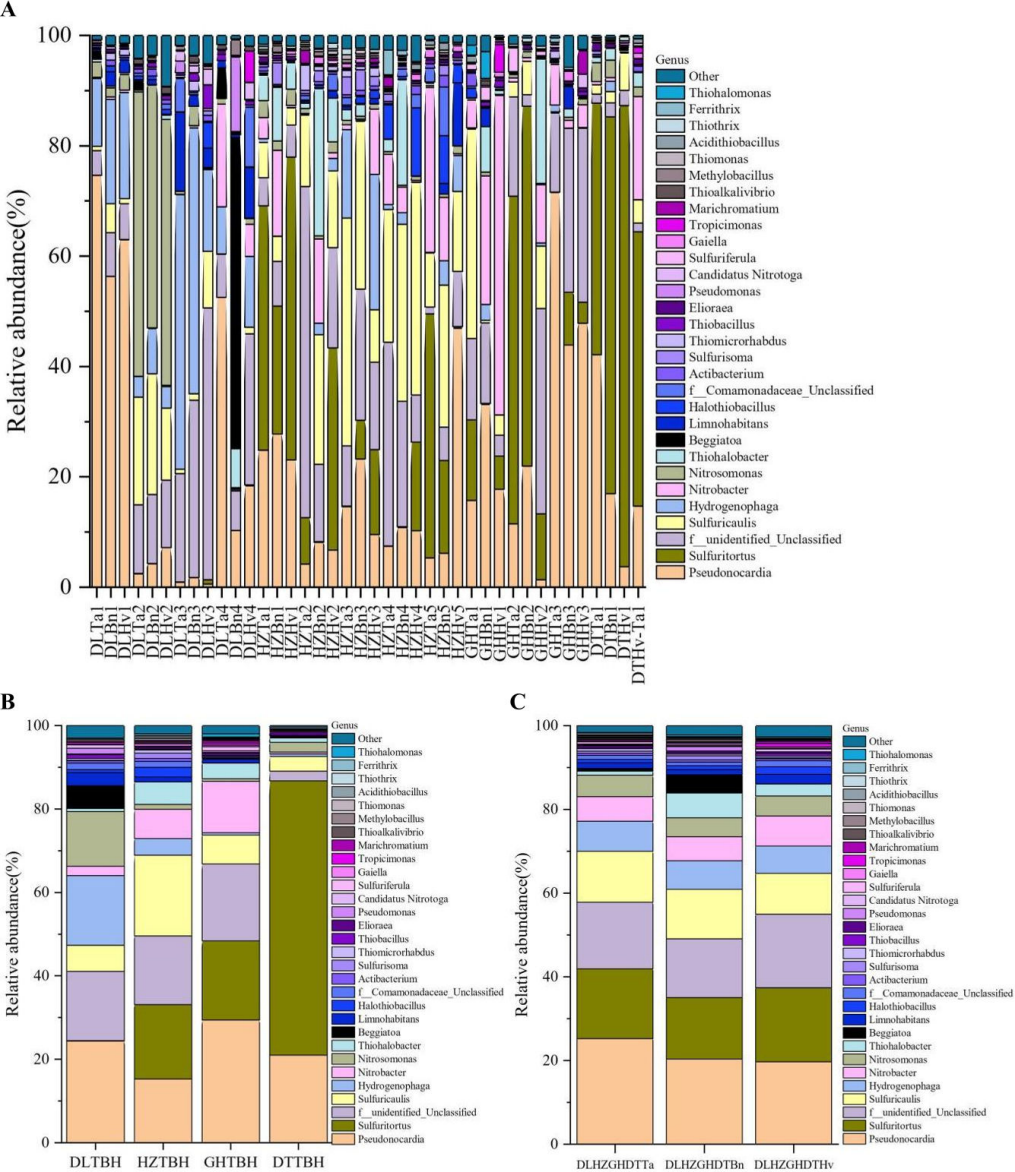
Distribution and relative abundances of the dominant genera in 40 soil samples (**A**), four region groups (**B**), and three crop types (**C**). The abscissa is the name of the soil sample. The ordinate is the relative abundance of different genera. The right color in the bar chart corresponds to the genus name of the following legend. Other presents the taxonomic name except for the 30 genera of relative abundance.

In total, 134 genera were identified in this study. The 10 common genera widely distributed in all 40 samples were *Pseudonocardia*, *Sulfuritortus*, *Sulfuricaulis*, *Nitrosomonas*, *Thiohalobacter*, *Nitrobacter*, *Thiobacillus*, *Elioraea*, *Thioalkalivibrio*, and *Thiomonas* (Fig. 10A). The genera *Pseudonocardia*, *Sulfuritortus*, *Sulfuricaulis*, *Nitrosomonas*, *Hydrogenophaga*, and unclassified genera were predominant, with the relative abundances of 17.76%–74.67%, 36.63%–83.55%, 25.74%–41.30%, 44.03%–51.56%, 24.56%–49.77%, and 27.39%–59.96%, respectively, in 11, 10, 6, 3, 3, and 5 soil samples, respectively. Additionally, the genus *Beggiatoa* was characteristic of DLBn4 (56.37%) soil, whereas *Thiohalobacter* was characteristic of HZBn2 (26.66%) soil. The genus composition in the four regions showed greater variation than in the three crop soils (Fig. 10B and C). In the DL soils, *Pseudonocardia* (22.36%), an unclassified genus (16.61%), *Hydrogenophaga* (16.72%), and *Nitrosomonas* (13.11%) were the major genera. The dominant genera in the GH soils were *Pseudonocardia* (29.40%), *Sulfuritortus* (18.97%), unclassified bacteria (18.46%), and *Nitrobacter* (12.34%). The predominant genera were *Sulfuricaulis* (19.36%), *Sulfuritortus* (17.84%), unclassified bacteria (16.47%), and *Pseudonocardia* (15.27%) in the HZ soils and *Sulfuritortus* (65.79%) and *Pseudonocardia* (20.96%) in the DT soils. The four genera *Pseudonocardia*, *Sulfuritortus*, unclassified bacteria, and *Sulfuricaulis* were dominant across all three crop soils, with relative abundances of 19.72%–25.22%, 14.66%–17.65%, 14.10%–17.62%, and 9.76%–12.16%, respectively.

### Differences in the marked taxa of soil CO_2_-assimilating bacteria

**Fig 11:**
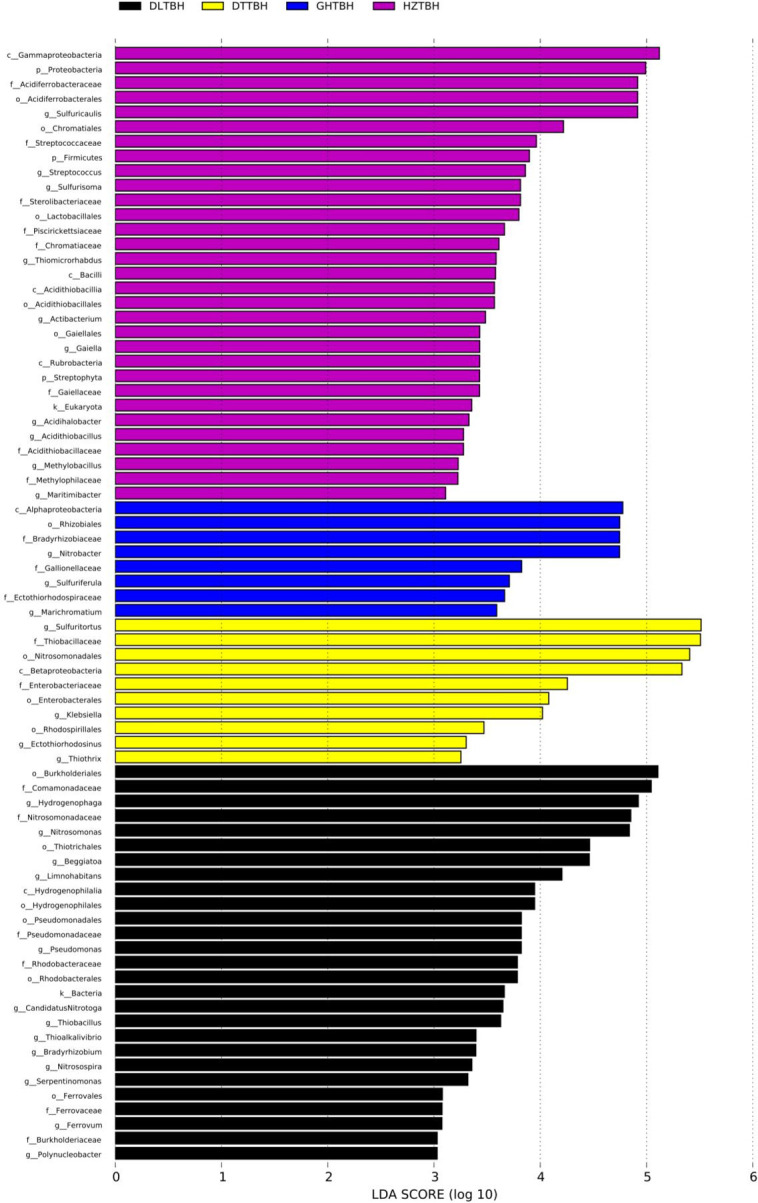
Histogram presenting the phylogenetic distribution of CO_2_-assimilating bacterial taxa in four region groups based on LEfSe analysis.

Autotrophic microbial community features in the soils that were significantly different between groups were identified using LEfSe analysis. A total of 31, 27, 10, and 8 significant biomarkers were identified in the four regions collected from the HZ, DL, DT, and GH, respectively (Fig. 11). The HZ group was enriched with bacterial lineages at different taxonomic levels, and included the phyla Proteobacteria, Firmicutes, and Streptophyta; classes Gammaproteobacteria, Bacilli, Acidithiobacillia and Rubrobacteria; orders Chromatiales, Acidiferrobacterales, Lactobacillales, Acidithiobacillales, and Gaiellales; families Acidiferrobacteraceae, Streptococcaceae, Sterolibacteriaceae, Piscirickettsiaceae, Chromatiaceae, Gaiellaceae, Acidithiobacillaceae, and Methylophilaceae; and genera *Sulfuricaulis*, *Streptococcus*, *Sulfurisoma*, *Thiomicrorhabdus*, *Actibacterium*, *Gaiella*, *Acidihalobacter*, *Acidithiobacillus*, *Methylobacillus*, and *Maritimibacter*.

Bacterial lineages such as class Alphaproteobacteria; order Rhizobiales; families Bradyrhizobiaceae, Gallionellaceae, and Ectothiorhodospiraceae; and genera *Nitrobacter*, *Sulfuriferula*, and *Marichromatium* were enriched in the GH group. Bacterial lineages including class Betaproteobacteriaceae; orders Nitrosomonadales, Enterobacterales, and Rhodospirillales; families Thiobacillaceae and Enterobacteriaceae; and genera *Sulfuritortus*, *Klebsiella*, *Ectothiorhodosinus*, and *Thiothrix* were enriched in the DT group. The DL soil was enriched with bacterial lineages such as class Hydrogenophilalia; orders Thiotrichales, Hydrogenophilales, Pseudomonadales, Rhodobacterales, and Ferrovales; families Comamonadaceae, Nitrosomonadaceae, Pseudomonadaceae, Rhodobacteraceae, Ferrovaceae, and Burkholderiaceae; and genera *Hydrogenophaga*, *Nitrosomonas*, *Beggiatoa*, *Limnohabitans, Pseudomonas*, *Candidatus Nitrotoga*, *Thiobacillus*, *Thioalkalivibrio*, *Bradyrhizobium*, *Nitrosospira*, *Serpentinomonas*, *Ferrovum*, and *Polynucleobacter*. The variation of enriched autotrophs in each region was identified at the class, order, family, and genus levels (Fig. S5). The relative abundances of bacterial lineages exhibited the most differences in the planted crop across different regions (Fig. S6). However, no significant biomarkers were detected in any of the three crop groups.

### Region and crop type affected the abundance of autotrophic bacteria and RubisCO activity

**Fig 12:**
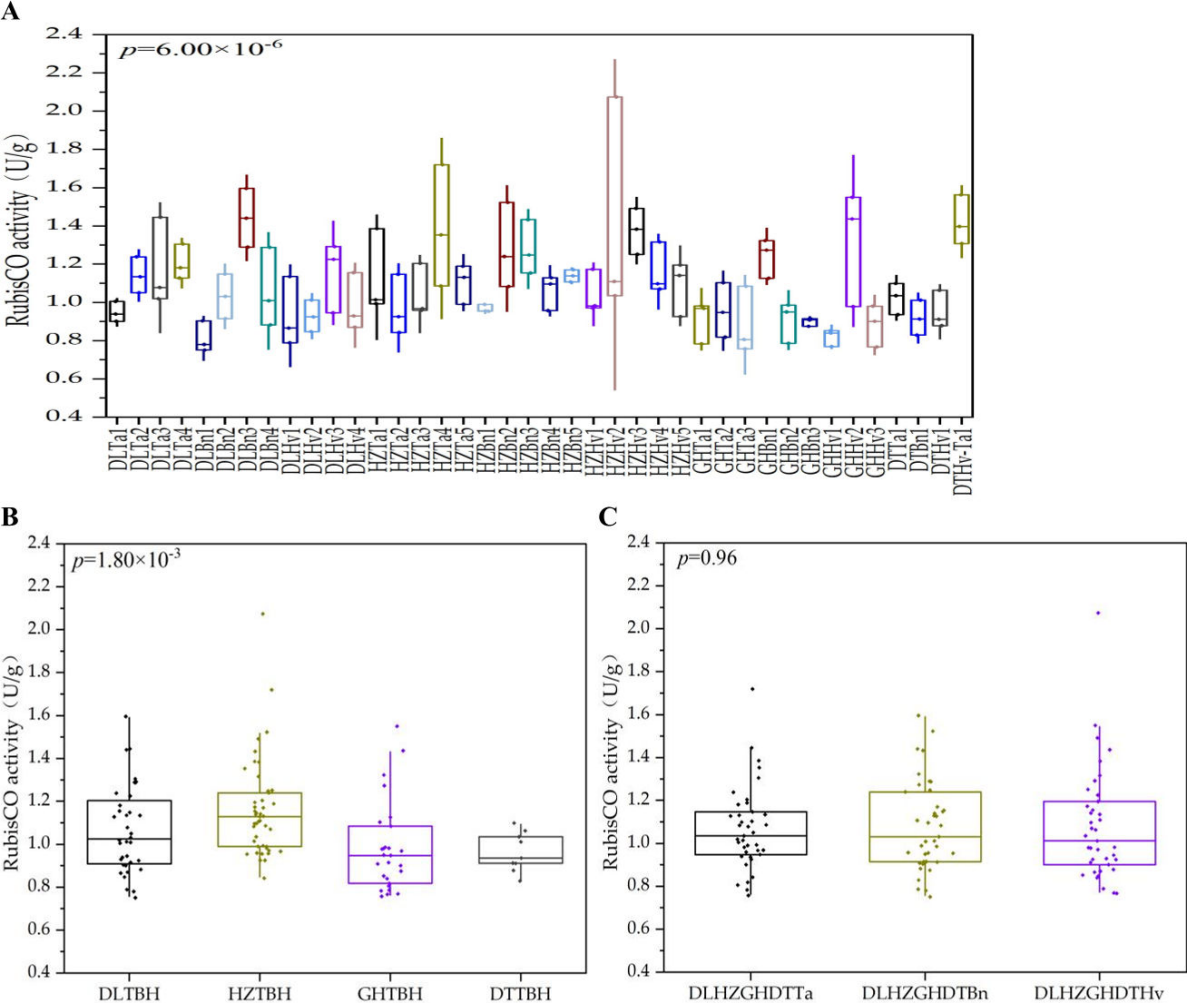
Soil RubisCO activity in 40 samples (**A**), four region groups (**B**), and three crop types (**C**). The abscissa is the name of the soil sample. DLTBH: DL region soils; HZTBH: HZ region soils; GHTBH: GH region soils; DTTBH: DT region soils; DLHZGHDTTa: wheat soils; DLHZGHDTBn: oilseed rape soils; and DLHZGHDTHv: barley soils.

Significant (*P* < 0.05, *P* < 0.001, Table 1) regional effects were detected for soil RubisCO activity and some autotrophic bacterial genera, such as *Sulfuritortus*, *Sulfuricaulis*, *Hydrogenophaga*, *Nitrobacter*, *Nitrosomonas*, *Thiohalobacter*, and *Beggiatoa*. In addition, some interactions were observed between region and crop type for *Sulfuritortus*, *Nitrobacter*, *Thiohalobacter*, and *Beggiatoa* (*P* < 0.05). The RubisCO activities of all the soils ranged from 0.81 ± 0.08 to 1.44 ± 0.15 U/g soil and were 1.94 times greater in the DLBn1 soil than in the DLBn3 soil (Fig. 12A). RubisCO activity in the HZ region was significantly greater than in the other three soil samples (*P* < 0.001) (Fig. 12B), with no significant difference among the three crop types (Fig. 12C).

### Relationships among selected autotrophic bacterial genera, indicators of *cbbL* gene, and soil properties

The correlations among the selected autotrophic bacterial genera, autotrophic bacterial properties, and selected soil factors were investigated using Pearson correlation analysis (Table 2). The relative abundance of *Sulfuritortus* was negatively correlated with the autotrophic bacterial Chao1 and Ace (*P* < 0.05), and Simpson, Shannon, and altitude (*P* < 0.01), but positively correlated with organic matter, total phosphate, and total sulfur levels (*P* < 0.05). *Hydrogenophaga* was positively associated with the Ace, Shannon, altitude, and ammonium nitrogen content (*P* < 0.05, *P* < 0.01), but negatively correlated with total phosphate (*P* < 0.05). In addition, *Limnohabitans* was the most positively correlated with Shannon and ammonium nitrogen, but *Pseudonocardia* was the most negatively correlated with RubisCO activity (*P* < 0.01) and ammonium nitrogen (*P* < 0.05). The abundances of both *Sulfuricaulis* and *Thiohalobacter* were positively correlated with total phosphate, whereas the abundance of *Beggiatoa* was negatively correlated with total phosphate (*P* < 0.05). The unclassified genera exhibited a positive correlation with Shannon, Simpson, altitude, and ammonium nitrogen, but a negative relationship with the soil water content, organic matter content, and effective phosphate (*P* < 0.05). No significant relationships were detected between the selected soil properties and the α-diversity indices of the *cbbL* gene (Table S7).

**TABLE 2:** Correlation analysis between selected autotrophic bacterial genera, indicators measured of *cbbL* gene and soil physicochemical properties

α-Diversity indices and soil properties	Pseudonocardia	Sulfuritortus	Unclassified genus	Sulfuricaulis	Hydrogenophaga	Nitrobacter	Nitrosomonas	Thiohalobacter	Beggiatoa	Limnohabitans
Ace	0.231	−0.372^*a*^	−0.088	0.118	0.323^*a*^	0.001	0.007	0.034	−0.182	0.200
Chao1	0.207	−0.368^*a*^	−0.084	0.125	0.314	0.019	0.001	0.058	−0.184	0.211
Shannon	0.087	−0.573^*b*^	0.338^*a*^	0.115	0.332^*a*^	−0.008	0.093	0.091	−0.275	0.344^*a*^
Simpson	−0.063	−0.590^*b*^	0.428^*b*^	0.172	0.230	0.080	0.127	0.115	−0.211	0.300
Altitude	−0.073	−0.489^*b*^	0.318^*a*^	−0.149	0.437^*b*^	−0.043	0.331^*a*^	−0.111	0.079	0.291
pH	0.000	−0.286	0.301	−0.071	−0.021	0.272	−0.029	−0.032	0.103	−0.015
Water content	−0.126	0.294	−0.315^*a*^	0.118	0.010	0.070	−0.271	0.041	0.095	−0.135
Organic matter	−0.197	0.379^*a*^	−0.329^*a*^	0.255	−0.185	0.144	−0.132	0.262	−0.215	−0.274
Ammonium nitrogen	−0.326^*a*^	−0.164	0.313^*a*^	−0.118	0.684^*b*^	−0.124	0.058	−0.160	−0.065	0.352^*a*^
Nitrate nitrogen	0.278	0.000	−0.127	0.168	−0.224	−0.074	−0.186	0.079	0.024	−0.253
Total phosphate	−0.121	0.317^*a*^	−0.271	0.393^*a*^	−0.313^*a*^	0.078	−0.093	0.347^*a*^	−0.351^*a*^	−0.242
Effective phosphate	−0.127	0.263	−0.323^*a*^	−0.183	0.032	0.223	0.220	0.094	−0.181	−0.041
Total sulfur	−0.075	0.355^*a*^	−0.289	0.139	−0.038	−0.087	−0.181	0.164	−0.156	−0.017
Effective sulfur	0.034	0.117	–0.146	0.114	−0.173	−0.205	−0.157	0.044	0.139	0.269
RubisCO activity	−0.434^*b*^	−0.088	0.213	0.197	0.246	0.047	−0.077	0.306	0.015	0.043

^*a*^ Correlation is significant at the 0.05 level.

^*b*^ Correlation is significant at the 0.01 level.

## DISCUSSION

The *cbbL* gene is a key gene that is usually used as an index for evaluating autotrophic potential. The present study showed variations in the diversity, structure, and activity of soil autotrophic CO_2_-fixing bacteria among four agricultural regions and three crop types. The effect of region on these changes was more evident than on the other crop groups.

### Differential diversity of soil autotrophic CO_2_-fixing bacteria between regions and crop types

The Chao1 and Shannon diversity indices of the three crop groups from all four regions did not significantly differ, indicating comparable bacterial diversity in the wheat, barley, and oilseed rape soils. Besides, the α-diversity indices were similar between barley and wheat soils from the same site. These results disagree with the findings of Yuan et al. (44), who reported that in the Pantang Agroecosystem, the species richness, Shannon-Wiener index, and evenness of *cbbL*-containing bacteria in rice-rice management soil were greater than those in rice-wheat and wheat-corn rotation soils. These inconsistent results may be related to geography. Whereas, in certain sampling locations, the Chao1 and Shannon diversity indices varied significantly between oilseed rape and other crop soils. The Shannon-Wiener index and Pielou index of *cbbL*-containing bacteria varied significantly among the four rice soils from four different regions (35). The Chao1 and Shannon diversity indices for wheat, oilseed rape, and barley soils from the four regions differed significantly. Additionally, the NMDS ordination showed dissimilar plots of the four regions that denoted variations in the bacterial community, and similar plots of the three crop soils from the four regions. A greater autotrophic microbial α-diversity was observed in the DL and HZ samples than in the GH and DT soils, suggesting distinct diversity in the four regions.

Generally, the autotrophic bacterial diversity varies with soil conditions, such as pH, total organic carbon, and nitrogen content (15, 45, 46). For instance, the Chao1 and Shannon indices of autotrophic bacteria at the study sites were negatively correlated with soil organic carbon and total nitrogen (31). In contrast, there was no significant relationship between the soil physiochemical parameters and the diversity of the *cbbL*-containing bacteria in the present study. This was also observed for different land use types (47). These results suggest that some soil properties do not affect the biodiversity of the *cbbL*-containing bacteria.

### Differential structure of soil autotrophic CO_2_-fixing bacteria between regions and crop types

Carbon fixation was genetically monitored using the *cbbL* gene marker, which encodes the key photosynthesis related enzyme RubisCO. The distribution and quantification of *cbbL* gene-containing bacteria in terrestrial ecosystems have largely focused on agricultural bulk soil (13, 26, 31, 34, 47–49). The results showed the number of shared and unique OTUs among the 40 soil samples, four regional groups, and three crop types. The four paddy soils from the four different regions had common dominant terminal restriction fragments (58 and 125 bp). In addition, some soils have common or unique dominant microbial populations (45). The predominant microbial populations in the four paddy soils from the four different regions varied, and most of these OTUs were distantly related to known sequences (35). Furthermore, our obtained sequences were mostly assigned to Proteobacteria and Actinobacteria, which commonly exist in farmland soils (12, 26, 33, 35, 46, 50). Long et al. (12) and Liu et al. (35) identified Proteobacteria and Actinobacteria as the dominant phyla in paddy soils from five sites and four sites in South China, respectively. In addition, our present study indicated a high diversity of autotrophic bacterial taxa in the four soil regions, which is consistent with previous findings (12).

The *cbbL* gene sequences at the genus level were assigned mainly to *Bradyrhizobium*, *Azospirillum*, *Rhodopseudomonas*, *Variovorax*, *Methylibium*, and *Pseudonocardia* (14, 16, 25, 37). The high abundances of the autotrophic bacterial dominant genera *Xanthobacter*, *Bradyrhizobium*, *Aminobacter*, and *Nitrosospira* were detected in maize soil (26). The relative percentages of the dominant autotrophic bacterial phyla, classes, and genera differed among the paddy soils from five sites in South China, in which the values for *Rhodoferax* varied from 0.006 to 0.15 (12). In the present study, the 10 most common genera among the samples were *Pseudonocardia*, *Sulfuritortus*, *Sulfuricaulis*, *Nitrosomonas*, *Thiohalobacter*, *Nitrobacter*, *Thiobacillus*, *Elioraea*, *Thioalkalivibrio*, and *Thiomonas*. The dominant genera *Pseudonocardia*, *Sulfuritortus*, *Sulfuricaulis*, and *Nitrosomonas* varied greatly among the four soil regions, and are involved in the geochemical cycling of sulfur and nitrogen.

Similarly, at the species level, the bacterial phylotypes exhibited diverse distribution patterns. The *cbbL* gene sequence obtained from groundnut rhizospheric soil contained the most *Ochrobactrum* and *Rhizobium* strains and included functional groups such as *R. leguminosarum*, *Bradyrhizobium japonicum*, *S. meliloti*, *O. anthropi*, *Acidithiomicrobium* sp., and *Rubrivivax gelatinosus*, which significantly contribute to the C or N cycles, and sulfur, CO-, and H_2_ oxidation (27). Tolli and King (14) observed distinct soil lithotrophic soil communities associated with land use and crop type (cotton and peanut) in agroecosystems. Yuan et al. (44) reported that *Mycobacterium* sp., *Rhodopseudomonas palustris*, *B. japonicum*, *Ralstonia eutropha*, and *Alcaligenes eutrophus* were detected in all soil samples and composed the majority of the *cbbL*-containing bacterial community under different land uses, whereas *Thiobacillus denitrificans*, *Nitrobacter winogradskyi*, and *N. vulgaris* composed only a small part of the total microbial community. In addition, the *cbbL*-containing bacterial community composition in double rice soil differed from that in wheat-corn and rice-wheat rotation soils from the Pantang Agroecosystem (44). Similarly, Wu et al. (29) reported that *R. palustris*, *B. japonicum*, *R. gelatinosus*, and *R. eutropha* were distributed in rice and corn soils from different geographical regions in China, and that these species could fix CO_2_. However, *Acidiphilium multivorum* and *Synechococcus* sp. were detected only in corn soil, and the *T. alkaliphila* strain ALgr 6 sp. was found only in rice soils. *Pseudonocardia* sp., *S. calidifontis*, *S. limicola*, *Nitrobacter* sp., *T. thiocyanaticus*, *N. marina*, *Elioraea* sp., *N. hamburgensis*, and *Thiobacillus* sp. were detected in the soils investigated. *N. marina*, *Elioraea* sp. YIM 72297, *N. hamburgensis*, and *Thiobacillus* sp. 63–78 composed only a small portion of the total microbial community. In particular, the *cbbL*-containing bacterial community compositions of the four regions were diverse, but the *cbbL*-containing populations of the three crop soils were similar. Furthermore, differential indicator groups were present in the four soil regions but not in the three crop-type soils. The dominant microbial groups in this study were quite different from those in paddy soil in South China (12, 35) and rice and corn soils in distinct geographical regions (29). These findings indicate that the distribution of the microbial community varies among different geographic locations. Various bacterial populations containing *cbbL* were selected in response to these variables. The obtained *cbbL* clone sequences were not closely affiliated with known cultured autotrophic bacteria and showed close proximity to uncultured bacteria retrieved from differently managed agricultural systems, agroecosystems, arid and semiarid soils (34). At present, the ecological roles of novel soil lithotrophs remain largely unknown (Fig. 10). These taxa are affiliated with new CO_2_-fixing bacteria, that have not yet been explored. The abundance and diversity of soil autotrophic bacteria are key factors for carbon sequestration (15, 16, 31). Shifts in autotrophic microorganisms with diverse metabolic strategies and activities may cause changes in CO_2_ fixation functions. The current study demonstrated that changes in autotrophic bacterial community composition can accurately account for the variation in the soil C fixation rate compared to that in abundance and α-diversity.

In the present study, organic matter, total phosphate, total sulfur, and ammonium nitrogen were found to have positive or negative relationships with the abundances of *Sulfuritortus*, *Sulfuricaulis*, *Thiohalobacter*, *Limnohabitans*, *Pseudonocardia*, *Beggiatoa*, or *Hydrogenophaga*. Similarly, Yuan et al. (37) also reported that total nitrogen and soil organic carbon were significantly correlated with the composition of the *cbbL*-containing bacteria in four different land use soils. Moreover, the CO_2_-fixing bacterial community was significantly affected by seven selected soil factors, such as total organic carbon, total sulfur, and ammonium nitrogen (12).

### Differences in RubisCO activities between regions and crop types

RubisCO activities are greater in paddy soils than in upland soils, and RubisCO activities differ significantly among paddy soils in different geographical regions (29). Wu et al. (30) reported that the RubisCO activities differed among rice-rice, rice-rapeseed, and rapeseed-corn rotated soils, with the highest activities occurring in rice-rice cropping systems. In the present study, RubisCO activity likely differed across different regions, but these values were similar among the three crop types from the four countries. This inconsistency can be attributed to differences in the geographical and *cbbL*-containing bacterial community compositions. Future research using stable isotope tracking is warranted to verify the causal links among the soil bacterial community, RubisCO activity, and CO_2_ emissions.

### Conclusions

Similar *cbbL*-bearing bacterial communities were observed among the three crop soils based on Chao1 and Shannon diversity indices, ANOSIM, LEfSe analysis, and the relative abundances of dominant taxa. However, significant differences in the *cbbL*-carrying bacterial community were observed between the four site pairs, indicating the roles of both latitude and longitude in structuring agroecosystem CO_2_-assimilating bacterial communities. The effect of region on RubisCO activity was more evident than on RubisCO activity in crop groups. The composition of the autotrophic bacterial community was significantly related to several soil properties.

## Data Availability

The data presented in this study are available within the article. The raw sequences of the cbbL clone libraries were deposited in the NCBI Sequence Read Archive (SRA) database under accession number PRJNA1024570.

## SUPPLEMENTAL MATERIAL

Supplemental tables and figures spectrum.02693-24-s0001.docx Tables S1 to S7; Fig. S1 to S5.

ASM does not own the copyrights to Supplemental Material that may be linked to, or accessed through, an article. The authors have granted ASM a non-exclusive, world-wide license to publish the Supplemental Material files. Please contact the corresponding author directly for reuse.
