# Supplementary material for: Differential diversity and structure of autotrophs in agricultural soils of Qinghai Province
Source: Microbiol Spectr. 2025 Jan 8;13(2):e02693-24. doi: 10.1128/spectrum.02693-24 (PMC11792524; doi:10.1128/spectrum.02693-24)
Supplement: Supplemental tables and figures — Tables S1 to S7; Fig. S1 to S5. [file spectrum.02693-24-s0001.docx]

Table S1 Geographical information of the soil samples

| Soil | | Village | East longitude | North latitude | Altitude  (m) | Crop | Plant area (m^2^) |
| --- | --- | --- | --- | --- | --- | --- | --- |
| Dulan (DL) | DLBn1 | Xitanheshang | 98°03′40.64″ | 36°21′18.46″ | 3124 | oilseed rape | 4920 |
|  | DLTa1 | Xitanheshang | 98°03′49.15″ | 36°21′18.27″ | 3124 | wheat | 4472 |
|  | DLHv1 | Xitanheshang | 98°03′40.64″ | 36°21′18.46″ | 3124 | barley | 3904 |
|  | DLBn2 | Shangzhuang | 98°05′56.83″ | 36°16′41.07″ | 3221 | oilseed rape | 3810 |
|  | DLTa2 | Shangzhuang | 98°05′54.27″ | 36°16′40.03″ | 3223 | wheat | 2288 |
|  | DLHv2 | Shangzhuang | 98°05′54.27″ | 36°16′40.03″ | 3223 | barley | 4497 |
|  | DLBn3 | Shangxitai | 98°06′33.27″ | 36°14′12.07″ | 3262 | oilseed rape | 4719 |
|  | DLTa3 | Shangxitai | 98°06′34.39″ | 36°14′11.29″ | 3261 | wheat | 7617 |
|  | DLHv3 | Shangxitai | 98°06′35.96″ | 36°14′11.33″ | 3260 | barley | 3363 |
|  | DLBn4 | Zhongzhuang | 98°05′35.96″ | 36°02′18.36″ | 3100 | oilseed rape | 2510 |
|  | DLTa4 | Zhongzhuang | 98°05′18.28″ | 36°02′20.72″ | 3097 | wheat | 3196 |
|  | DLHv4 | Zhongzhuang | 98°05′15.14″ | 36°02′18.36″ | 3100 | barley | 2136 |
| Huzhu (HZ) | HZBn1 | Xishangen | 101°55′06.31″ | 37°00′31.38″ | 2891 | oilseed rape | 532 |
|  | HZTa1 | Xishangen | 101°55′08.79″ | 37°00′29.21″ | 2890 | wheat | 973 |
|  | HZHv1 | Xishangen | 101°55′05.94″ | 37°00′30.98″ | 2888 | barley | 818 |
|  | HZBn2 | Xiakou | 101°56′59.42″ | 37°01′45.13″ | 3010 | oilseed rape | 1935 |
|  | HZTa2 | Xiakou | 101°57′33.06″ | 37°02′21.37″ | 3054 | wheat | 812 |
|  | HZHv2 | Xiakou | 101°57′33.06″ | 37°02′21.34″ | 3055 | barley | 693 |
|  | HZBn3 | Donggou | 101°55′24.11″ | 37°01′43.38 | 2997 | oilseed rape | 3851 |
|  | HZTa3 | Donggou | 101°55′15.24″ | 37°01′49.82″ | 2988 | wheat | 2175 |
|  | HZHv3 | Donggou | 101°55′23.83″ | 37°01′43.15″ | 2996 | barley | 1615 |
|  | HZBn4 | Zhangjiazhuang | 101°53′11.21″ | 37°01′06.21″ | 2864 | oilseed rape | 709 |
|  | HZTa4 | Zhangjiazhuang | 101°53′11.71″ | 37°01′06.45″ | 2866 | wheat | 1212 |
|  | HZHv4 | Zhangjiazhuang | 101°53′11.17″ | 37°01′06.21″ | 2865 | barley | 1294 |
|  | HZBn5 | Dahualin | 102°06′13.55″ | 36°58′10.86″ | 3016 | oilseed rape | 650 |
|  | HZTa5 | Dahualin | 102°06′06.15″ | 36°57′51.95″ | 3018 | wheat | 1079 |
|  | HZHv5 | Dahualin | 102°06′08.23″ | 36°57′51.71″ | 3017 | barley | 618 |
| Gonghe  (GH) | GHBn1 | Yilangtang | 100°41′20.87″ | 36°20′44.34″ | 3057 | oilseed rape | 695 |
|  | GHTa1 | Yilangtang | 100°41′20.87″ | 36°20′44.34″ | 3057 | wheat | 600 |
|  | GHHv1 | Yilangtang | 100°41′20.87″ | 36°20′44.34″ | 3057 | barley | 143 |
|  | GHBn2 | Gouhou | 100°35′02.17″ | 36°23′21.32″ | 3205 | oilseed rape | 1348 |
|  | GHTa2 | Gouhou | 100°35′02.22″ | 36°23′21.33″ | 3206 | wheat | 466 |
|  | GHHv2 | Gouhou | 100°35′02.21″ | 36°23′16.54″ | 3200 | barley | 841 |
|  | GHBn3 | Tala | 99°57′52.20″ | 36°11′23.13″ | 3058 | oilseed rape | 4010 |
|  | GHTa3 | Tala | 99°58′48.16″ | 36°11′58.66″ | 3043 | wheat | 14342 |
|  | GHHv3 | Tala | 99°57′52.11″ | 36°11′23.21″ | 3061 | barley | 4085 |
| Datong (DT) | DTBn1 | Geda | 101°39′22.24″ | 37°03′08.69″ | 2695 | oilseed rape | 797 |
|  | DTTa1 | Geda | 101°39′22.20″ | 37°03′08.68″ | 2694 | wheat | 673 |
|  | DTHv1 | Geda | 101°39′27.12″ | 37°03′11.36″ | 2709 | barley | 86 |
|  | DTTa-Hv1 | Yaocao | 101°39′20.10″ | 37°04′10.24″ | 2748 | wheat and barley | 638 |


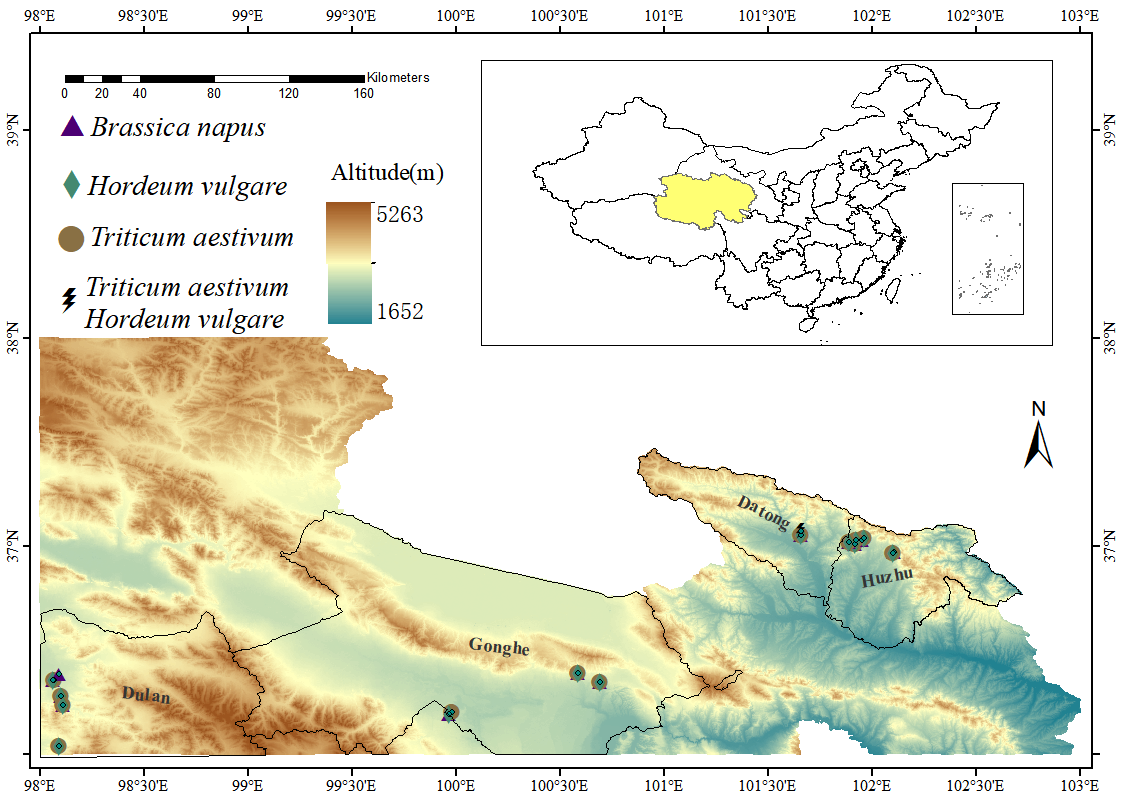


Figure S1 A map of the sampled locations.

Table S2 Alpha diversity indices of the *cbbL* gene in the DL region

| Soil | Ace | Chao1 | Shannon | Simpson | Goods coverage |
| --- | --- | --- | --- | --- | --- |
| DLBn1 | 2523.04±264.80^a^ | 2442.06±289.16^a^ | 6.30±0.87^a^ | 0.886±0.041^a^ | 0.979±0.001^f^ |
| DLBn2 | 1507.86±66.34^cd^ | 1473.27±77.06^cd^ | 5.89±0.34^abc^ | 0.935±0.019^a^ | 0.988±0.001^bcd^ |
| DLBn3 | 1556.73±132.33^cd^ | 1535.89±138.74^cd^ | 6.01±0.25^ab^ | 0.945±0.006^a^ | 0.987±0.002^cd^ |
| DLBn4 | 907.04±163.17^e^ | 915.29±198.06^e^ | 3.69±0.44^d^ | 0.762±0.065^b^ | 0.992±0.002^a^ |
| DLHv1 | 2197.00±545.61^ab^ | 2120.33±520.68^ab^ | 5.27±1.36^abc^ | 0.790±0.108^b^ | 0.981±0.004^ef^ |
| DLHv2 | 1492.88±325.52^cd^ | 1505.89±319.41^cd^ | 5.54±0.71^abc^ | 0.902±0.065^a^ | 0.988±0.003^bcd^ |
| DLHv3 | 1395.38±247.54^cde^ | 1391.79±229.46^cd^ | 6.28±0.37^a^ | 0.956±0.018^a^ | 0.989±0.002^abcd^ |
| DLHv4 | 1627.06±410.98^c^ | 1626.12±378.65^c^ | 5.45±0.72^abc^ | 0.918±0.021^a^ | 0.986±0.004^cd^ |
| DLTa1 | 1782.02±357.46^bc^ | 1717.57±318.60^bc^ | 4.83±1.22^cd^ | 0.761±0.120^b^ | 0.985±0.003^de^ |
| DLTa2 | 1119.14±270.66^de^ | 1104.72±237.42^de^ | 4.99±0.19^bc^ | 0.888±0.017^a^ | 0.991±0.002^ab^ |
| DLTa3 | 1303.32±248.00^cde^ | 1315.39±251.71^cde^ | 5.56±0.31^abc^ | 0.914±0.016^a^ | 0.990±.003^abc^ |
| DLTa4 | 1498.88±94.03^cd^ | 1482.58±106.39^cd^ | 5.79±0.15^abc^ | 0.927±0.005^a^ | 0.988±0.001^bcd^ |

Note: Data represent means ± standard deviation (SD). Means followed by different lowercase letters differ statistically (*p*<0.05).

Table S3 Alpha diversity indices of the *cbbL* gene in the GH region

| Soil | Ace | Chao1 | Shannon | Simpson | Goods coverage |
| --- | --- | --- | --- | --- | --- |
| GHBn1 | 1545.01±142.51^a^ | 1518.81±162.83^a^ | 6.17±0.40^a^ | 0.944±0.022^a^ | 0.988±0.002^d^ |
| GHBn2 | 768.18±228.45^cd^ | 771.72±239.73^cd^ | 3.93±1.24^b^ | 0.797±0.118^a^ | 0.994±0.002^ab^ |
| GHBn3 | 1020.37±338.48^abcd^ | 1036.16±325.21^abcd^ | 5.28±0.95^ab^ | 0.909±0.041^a^ | 0.992±0.003^abc^ |
| GHHv1 | 1321.85±421.55^ab^ | 1349.34±465.93^ab^ | 4.94±0.42^ab^ | 0.885±0.009^a^ | 0.989±0.004^cd^ |
| GHHv2 | 602.98±279.67^d^ | 616.63±286.44^d^ | 4.07±1.42^b^ | 0.810±0.160^a^ | 0.996±0.002^a^ |
| GHHv3 | 697.11±459.40^d^ | 699.98±448.32^cd^ | 4.66±1.43^ab^ | 0.874±0.103^a^ | 0.994±0.004^a^ |
| GHTa1 | 1238.14±393.50^abc^ | 1220.92±419.61^abc^ | 4.45±1.35^ab^ | 0.816±0.103^a^ | 0.990±0.004^bcd^ |
| GHTa2 | 765.95±134.32^cd^ | 753.64±148.44^cd^ | 3.95±1.11^b^ | 0.766±0.167^a^ | 0.994±0.001^ab^ |
| GHTa3 | 896.73±241.91^bcd^ | 892.20±249.53^bcd^ | 4.68±0.77^ab^ | 0.862±0.097^a^ | 0.993±0.002^abc^ |

Note: Data represent means ± standard deviation (SD). Means followed by different lowercase letters differ statistically (*p*<0.05).

Table S4 Alpha diversity indices of the *cbbL* gene in the HZ region

| Soil | Ace | Chao1 | Shannon | Simpson | Goods coverage |
| --- | --- | --- | --- | --- | --- |
| HZBn1 | 2059.31±273.63^a^ | 2042.74±293.62^a^ | 6.23±0.35^ab^ | 0.940±0.007^a^ | 0.983±0.003^g^ |
| HZBn2 | 1524.81±160.37^cdefg^ | 1605.83±158.99^bcd^ | 5.96±0.37^abcd^ | 0.944±0.013^a^ | 0.987±0.002^bcde^ |
| HZBn3 | 1066.51±186.92^h^ | 1033.04±177.00^f^ | 4.93±0.65^ef^ | 0.904±0.030^abc^ | 0.992±0.002^a^ |
| HZBn4 | 1898.06±262.15^ab^ | 1890.04±230.12^ab^ | 6.14±0.23^abcd^ | 0.947±0.005^a^ | 0.984±0.003^fg^ |
| HZBn5 | 1266.82±44.59^fgh^ | 1296.14±82.22d^ef^ | 5.25±0.62^def^ | 0.906±0.053^abc^ | 0.990±0.001^ab^ |
| HZHv1 | 1334.10±195.50^efgh^ | 1301.03±166.71^def^ | 4.80±0.78^f^ | 0.837±0.070^cd^ | 0.989±0.002^abc^ |
| HZHv2 | 1787.37±139.40^abcd^ | 1777.00±144.73^abc^ | 5.38±0.12^bcdef^ | 0.868±0.013^abcd^ | 0.985±0.001^defg^ |
| HZHv3 | 1652.99±80.61^bcde^ | 1656.61±73.10^bc^ | 5.77±0.32^abcde^ | 0.929±.018^ab^ | 0.986±0.001^cdef^ |
| HZHv4 | 1486.11±349.27^defg^ | 1473.75±375.46^cde^ | 4.99±0.54^ef^ | 0.886±0.035^abcd^ | 0.988±0.003^bcd^ |
| HZHv5 | 1815.11±52.39^abcd^ | 1846.68±82.57^ab^ | 6.63±0.29^a^ | 0.962±0.007^a^ | 0.985±0.001^defg^ |
| HZTa1 | 1879.13±152.37^abc^ | 1892.18±186.22^ab^ | 5.30±0.78^cdef^ | 0.819±0.072^de^ | 0.984±0.001^efg^ |
| HZTa2 | 1482.65±297.12^defg^ | 1482.99±303.93^cde^ | 6.22±0.53^ab^ | 0.953±0.014^a^ | 0.988±0.003^bcd^ |
| HZTa3 | 1657.09±139.94^bcde^ | 1623.23±117.72^bcd^ | 5.30±0.53^cdef^ | 0.852±0.077^bcd^ | 0.986±0.001^defg^ |
| HZTa4 | 1597.52±106.01^bcdef^ | 1628.95±72.66^bcd^ | 6.15±0.57^abc^ | 0.948±0.024^a^ | 0.987±0.001^bcdef^ |
| HZTa5 | 1176.72±372.71^gh^ | 1173.15±324.02^ef^ | 3.68±0.75^g^ | 0.736±0.125^e^ | 0.990±0.003^ab^ |

Note: Data represent means ± standard deviation (SD). Means followed by different lowercase letters differ statistically (*p*<0.05).

Table S5 Alpha diversity indices of the *cbbL* gene in the DT region

| Soil | Ace | Chao1 | Shannon | Simpson | Goods coverage |
| --- | --- | --- | --- | --- | --- |
| DTBn1 | 1118.13±242.35^a^ | 1140.79±339.40^a^ | 5.27±0.38^a^ | 0.915±0.016^a^ | 0.991±0.002^a^ |
| DTHv1 | 732.32±339.08^a^ | 765.90±295.60^a^ | 2.54±0.37^b^ | 0.551±0.040^b^ | 0.994±0.003^a^ |
| DTTa1 | 1079.63±209.02^a^ | 1094.78±243.03^a^ | 5.06±1.15^a^ | 0.831±0.121^a^ | 0.992±0.002^a^ |

Note: Data represent means ± standard deviation (SD). Means followed by different lowercase letters differ statistically (*p*<0.05).

| **A**  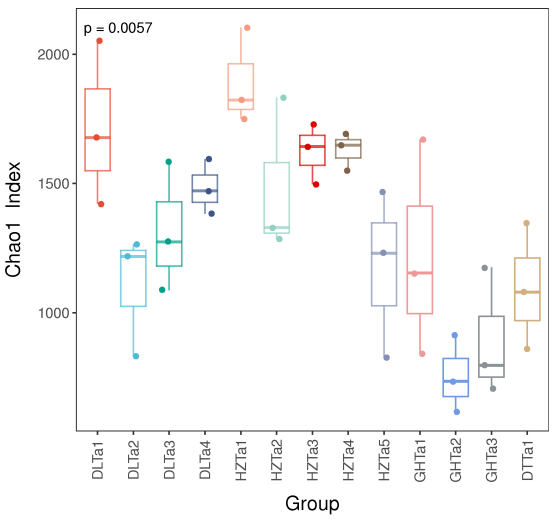 | **B**  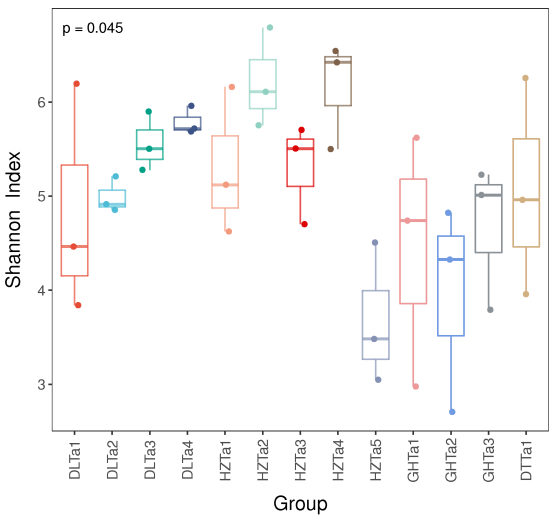 |
| --- | --- |
| **C**  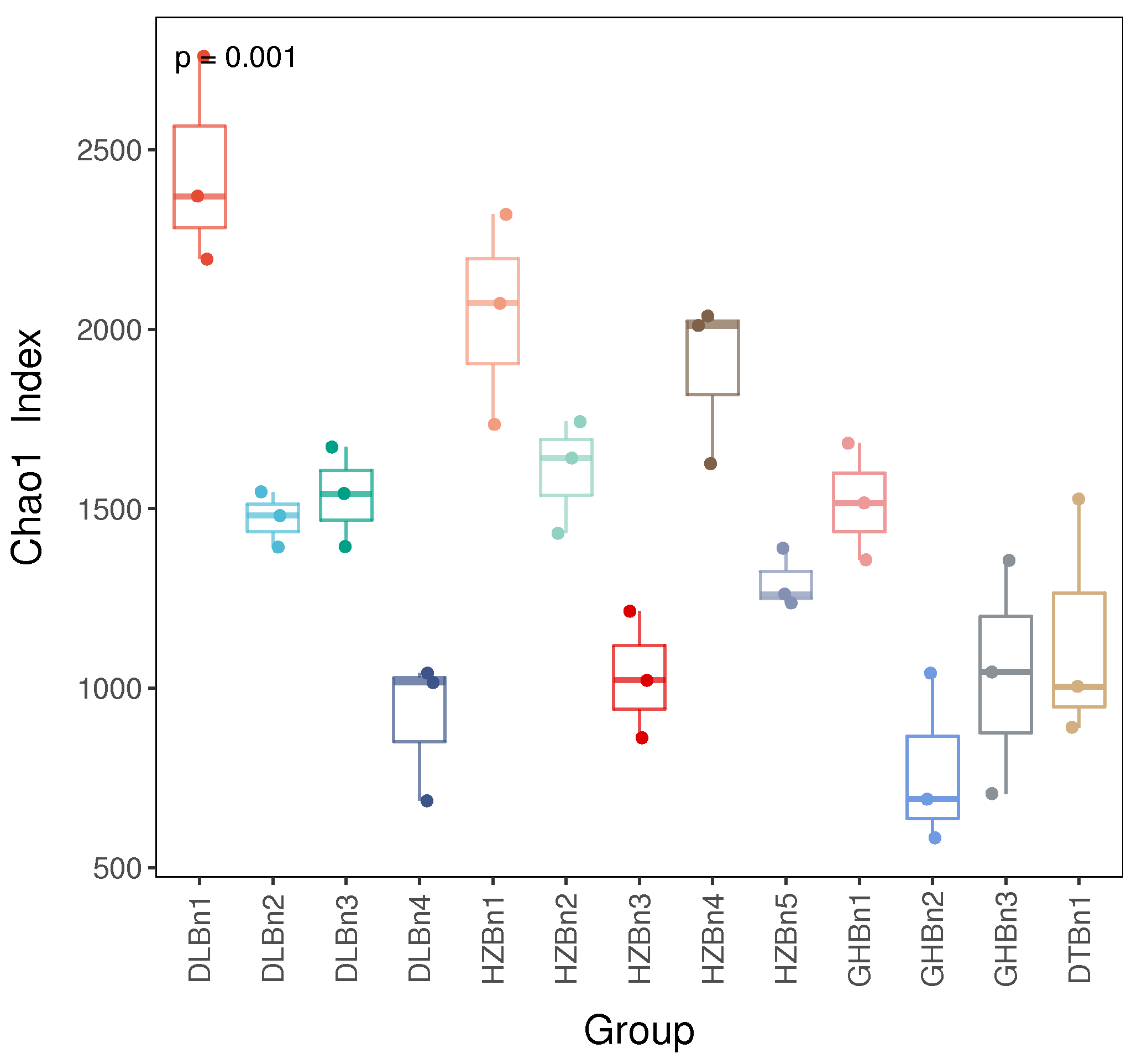 | **D**  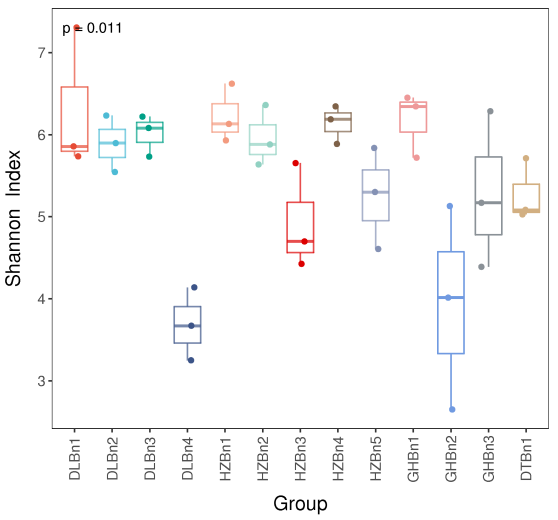 |
| **E**  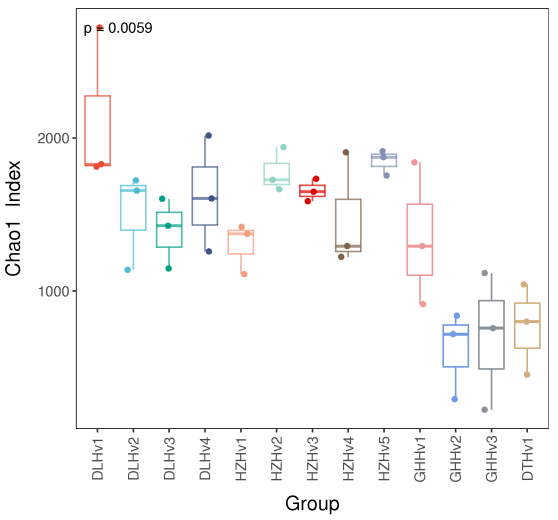 | **F**  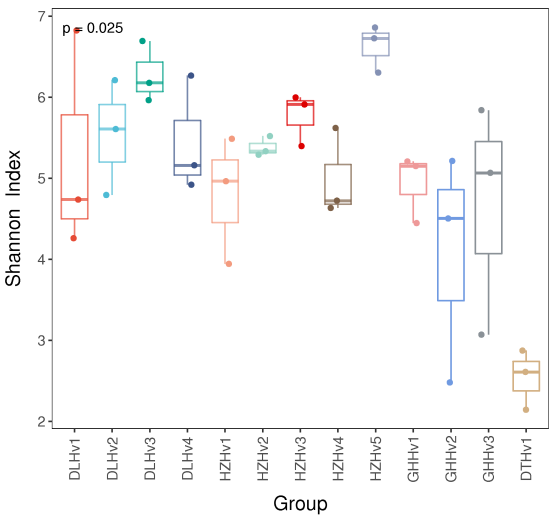 |

Figure S2 Chao1 and Shannon diversity indices of each crop soil (A and B: wheat, C and D: oilseed rape, E and F: barley).

| **A**  **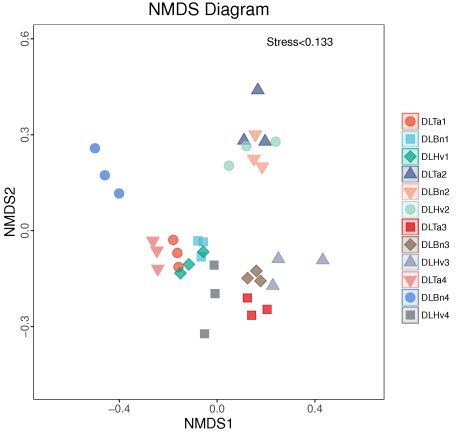** | **B**  **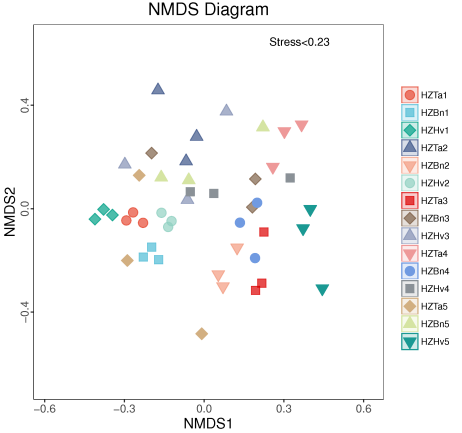** |
| --- | --- |
| **C**  **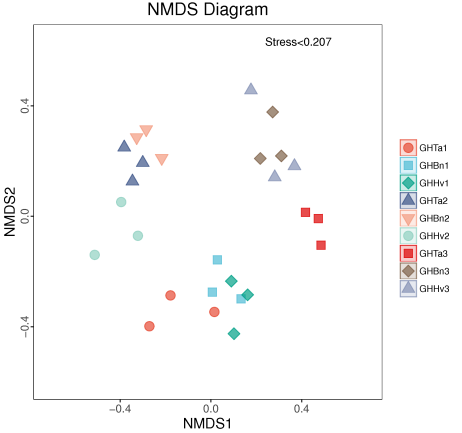** | **D**  **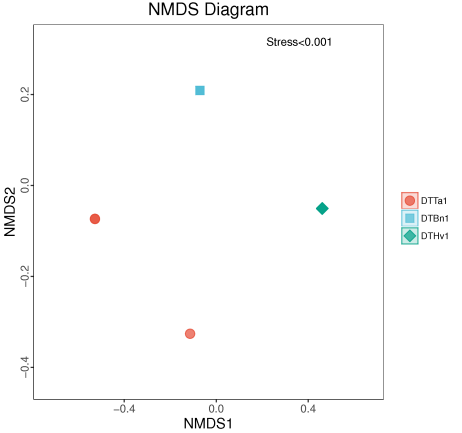** |

Figure S3 Nonmetric multidimensional scaling (NMDS) ordination pattern of the *cbbL*-containing bacterial community composition in the each region (A: DL, B: HZ, C: GH, D: DT).

| **A**  **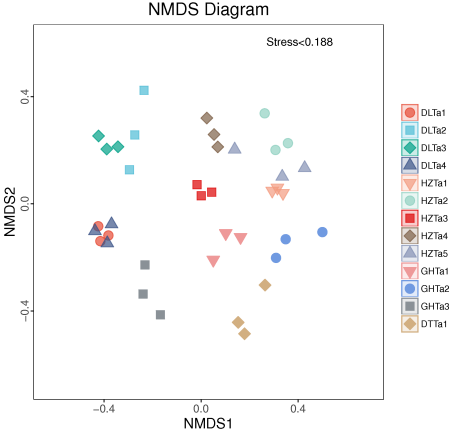** | **B**  **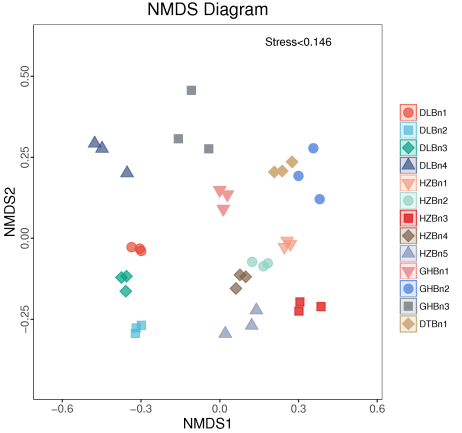** |
| --- | --- |
| **C**  **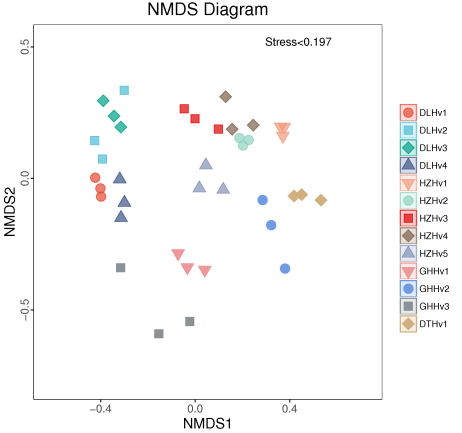** |  |

Figure S4 Nonmetric multidimensional scaling (NMDS) ordination pattern of the *cbbL*-containing bacterial community composition in each crop soil (A: wheat, B: oilseed rape, C: barley).

| **A**  **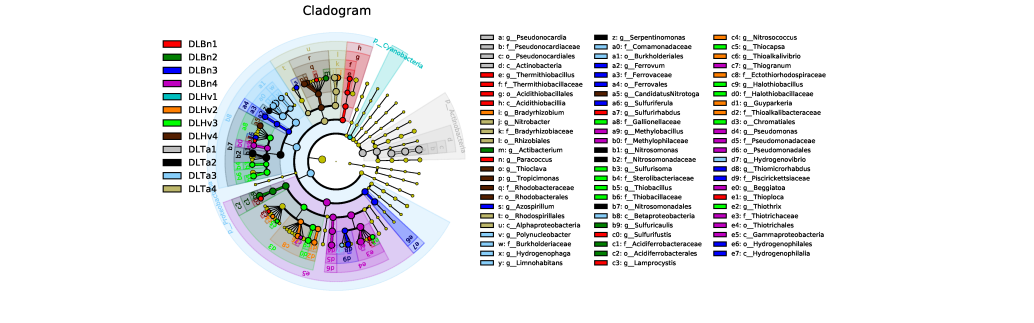** |
| --- |
| **B**  **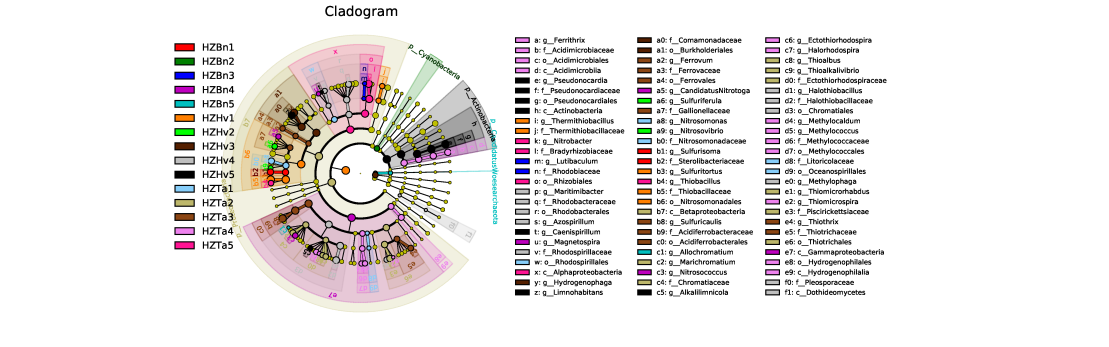** |
| **C**  **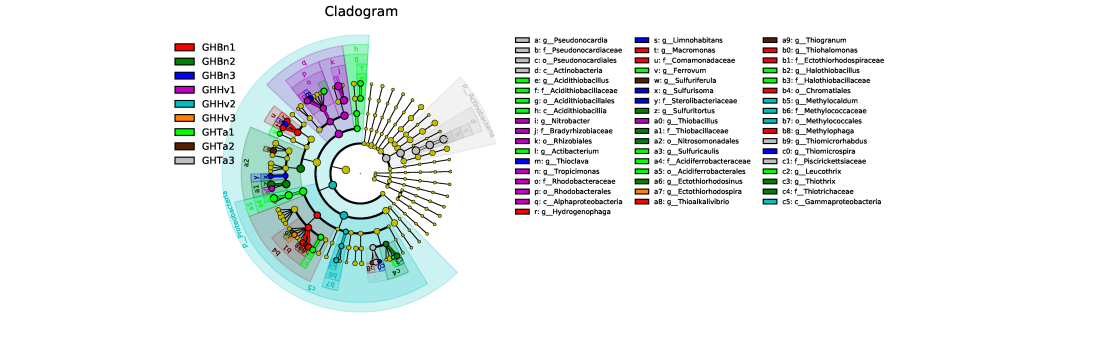** |
| **D**  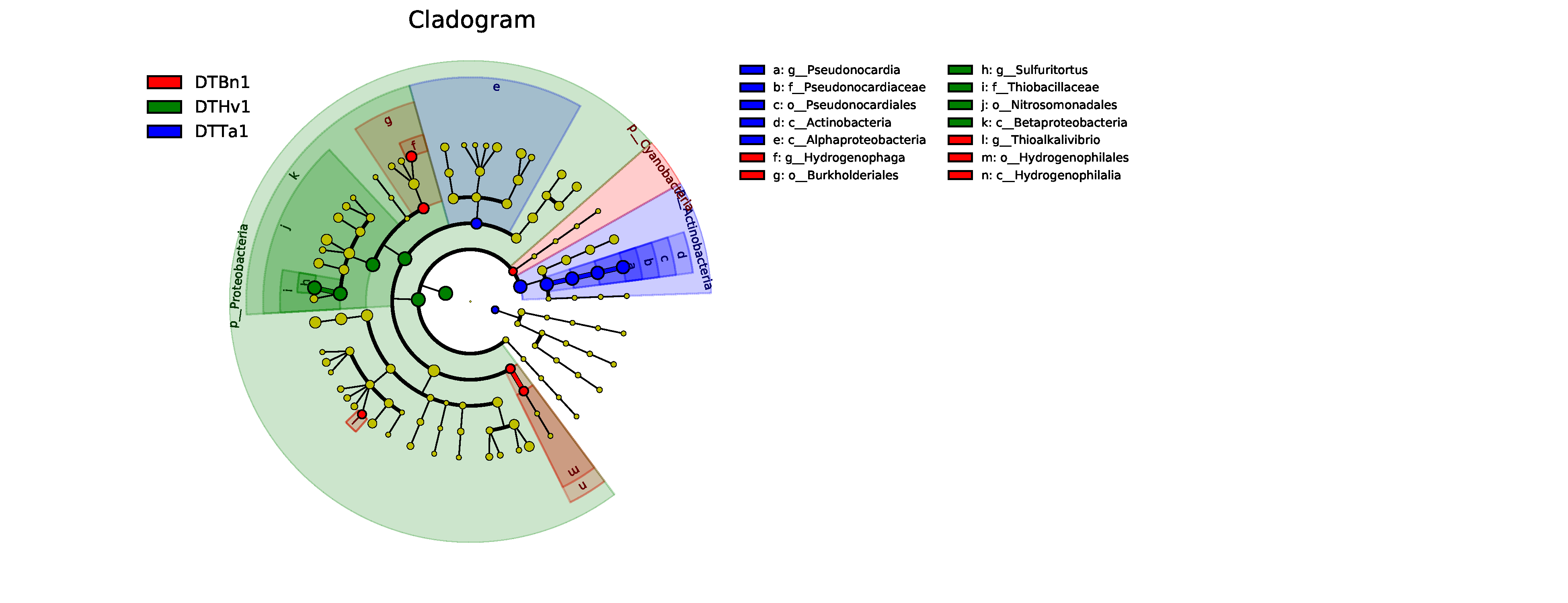 |

Figure S5 Cladogram presenting the phylogenetic distribution of CO_2_-assimilating bacterial taxa in each region (A: DL, B: HZ, C: GH, D: DT) based on LEfSe analysis.

| **A**  **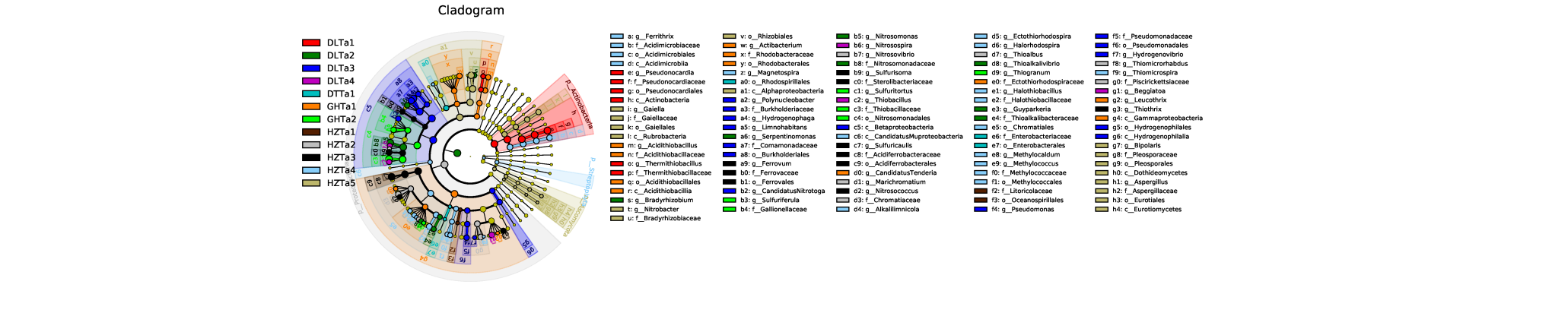** |
| --- |
| **B**  **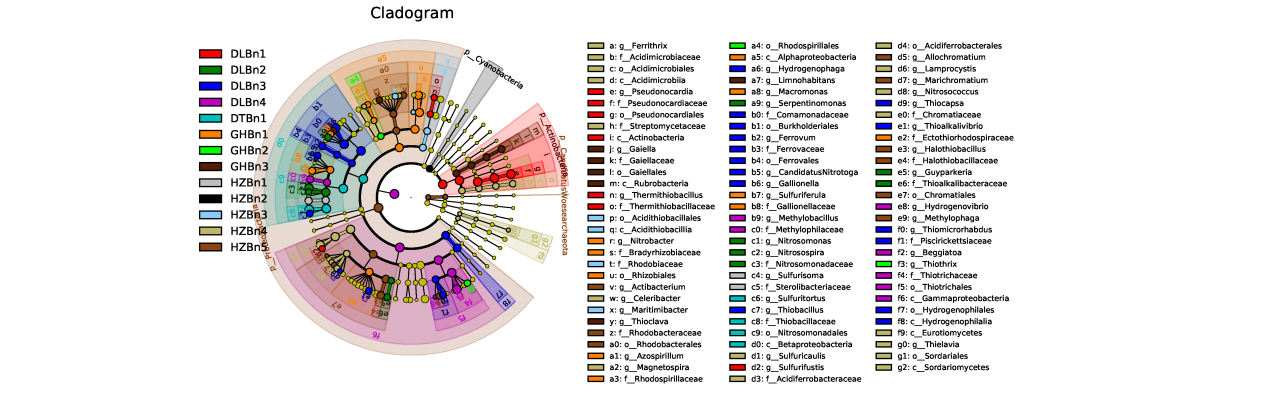** |
| **C**  **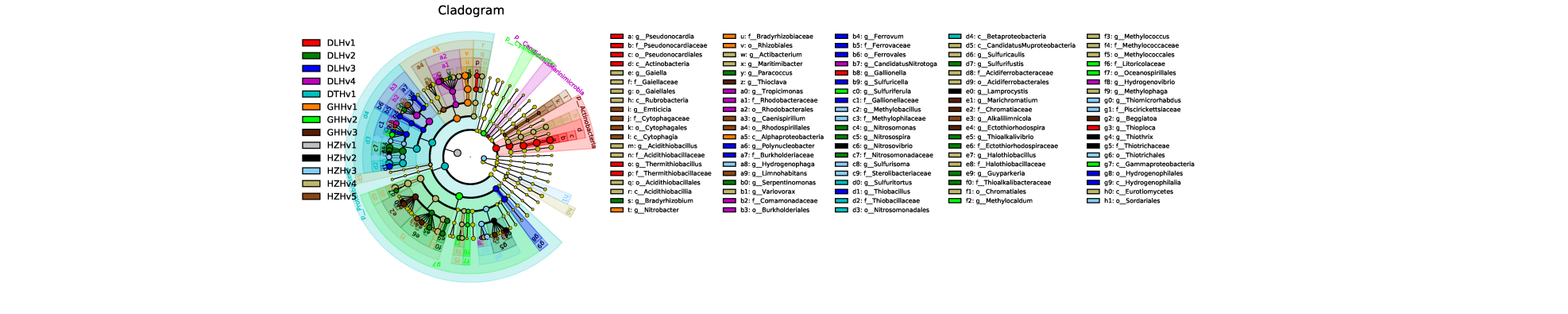** |

Figure S6 Cladogram presenting the phylogenetic distribution of CO_2_-assimilating bacterial taxa in each crop group (A: wheat, B: oilseed rape, C: barley) based on LEfSe analysis.

Table S6 Physicochemical properties of the soil samples

| Soil sample | pH | Water content  (%) | Organic matter  (g/kg) | Ammonium nitrogen  (mg/kg) | Nitrate nitrogen  (mg/kg) | Total phosphate  (g/kg) | Effective phosphate  (mg/kg) | Total sulfur  (mg/kg) | Effective sulfur  (mg/kg) |
| --- | --- | --- | --- | --- | --- | --- | --- | --- | --- |
| DLBn1 | 8.08±0.04 | 0.087±0.001 | 15.42±0.13 | 3.30±0.40 | 13.23±0.20 | 0.864±0.015 | 28.75±0.79 | 315.38±4.63 | 15.15±0.84 |
| DLTa1 | 8.39±0.02 | 0.082±0.006 | 13.75±0.60 | 4.07±0.23 | 8.42±0.17 | 0.868±0.021 | 13.52±0.76 | 514.93±19.04 | 12.01±0.98 |
| DLHv1 | 8.16±0.04 | 0.059±0.000 | 16.89±0.55 | 2.85±0.22 | 2.88±0.63 | 0.452±0.019 | 11.83±1.20 | 195.01±25.93 | 19.24±1.78 |
| DLBn2 | 8.28±0.01 | 0.168±0.041 | 18.82±1.54 | 4.39±0.17 | 3.75±0.64 | 0.766±0.010 | 16.34±1.93 | 346.53±13.21 | 18.47±1.71 |
| DLTa2 | 8.10±0.04 | 0.124±0.007 | 18.20±1.09 | 4.13±0.21 | 2.36±0.23 | 0.890±0.022 | 34.14±2.60 | 299.34±1.81 | 9.68±0.80 |
| DLHv2 | 8.24±0.08 | 0.111±0.004 | 16.20±0.39 | 3.78±0.12 | 3.81±0.31 | 0.750±0.023 | 28.77±1.02 | 191.91±25.29 | 19.27±1.21 |
| DLBn3 | 8.19±0.12 | 0.195±0.006 | 17.93±0.42 | 9.22±0.15 | 2.30±1.48 | 0.721±0.048 | 21.52±0.44 | 330.95±12.84 | 19.28±1.44 |
| DLTa3 | 8.18±0.01 | 0.186±0.013 | 12.92±0.42 | 7.28±0.24 | 1.61±0.25 | 0.637±0.032 | 16.35±1.58 | 471.90±34.64 | 15.38±0.64 |
| DLHv3 | 8.30±0.01 | 0.171±0.031 | 13.52±0.15 | 6.79±0.07 | 3.11±0.39 | 0.544±0.034 | 16.04±0.76 | 312.94±12.18 | 18.44±0.99 |
| DLBn4 | 8.38±0.04 | 0.088±0.003 | 10.66±0.70 | 3.28±0.17 | 7.61±0.18 | 0.367±0.006 | 3.97±0.56 | 210.83±12.38 | 30.38±0.93 |
| DLTa4 | 8.70±0.07 | 0.068±0.001 | 8.10±0.96 | 3.33±0.24 | 16.23±0.44 | 0.229±0.003 | 2.27±0.23 | 483.72±20.72 | 17.78±0.97 |
| DLHv4 | 8.24±0.04 | 0.091±0.002 | 10.62±0.79 | 3.71±0.35 | 5.47±0.64 | 0.574±0.008 | 21.67±1.52 | 259.29±17.02 | 22.71±0.47 |
| HZBn1 | 7.71±0.06 | 0.112±0.009 | 44.93±0.82 | 3.17±0.12 | 9.78±0.71 | 1.206±0.031 | 22.04±0.74 | 457.90±25.46 | 29.61±2.05 |
| HZTa1 | 8.04±0.04 | 0.176±0.013 | 37.56±0.95 | 3.38±0.27 | 14.88±1.45 | 1.124±0.058 | 34.60±0.19 | 232.77±14.85 | 23.63±0.74 |
| HZHv1 | 7.61±0.02 | 0.142±0.027 | 37.28±1.58 | 2.72±0.19 | 14.83±0.50 | 1.078±0.090 | 20.29±1.97 | 837.35±24.59 | 29.39±2.06 |
| HZBn2 | 7.89±0.08 | 0.219±0.006 | 36.41±0.71 | 2.92±0.21 | 6.64±0.66 | 1.434±0.052 | 13.52±1.16 | 342.07±10.80 | 25.89±1.76 |
| HZTa2 | 8.25±0.01 | 0.140±0.006 | 17.82±0.06 | 3.94±0.10 | 8.42±0.73 | 0.843±0.022 | 7.10±0.78 | 125.31±4.60 | 19.65±1.95 |
| HZHv2 | 8.02±0.01 | 0.200±0.019 | 42.58±1.76 | 2.69±0.09 | 16.50±0.36 | 1.039±0.014 | 16.74±0.87 | 870.44±6.85 | 43.50±0.53 |
| HZBn3 | 7.56±0.04 | 0.223±0.008 | 41.49±0.65 | 2.99±0.33 | 9.88±0.16 | 1.057±0.068 | 4.03±0.37 | 718.95±26.10 | 31.68±0.83 |
| HZTa3 | 8.03±0.01 | 0.179±0.004 | 30.14±0.23 | 3.16±0.26 | 13.47±1.13 | 0.966±0.029 | 11.63±0.72 | 146.89±27.24 | 7.33±0.26 |
| HZHv3 | 7.99±0.05 | 0.206±0.028 | 44.22±1.07 | 3.52±0.16 | 11.63±0.21 | 1.012±0.060 | 24.23±0.81 | 879.21±7.53 | 22.86±2.08 |
| HZBn4 | 8.40±0.02 | 0.156±0.012 | 32.29±0.31 | 2.62±0.03 | 14.23±0.56 | 1.034±0.024 | 15.52±3.12 | 891.17±17.81 | 31.28±1.93 |
| HZTa4 | 8.78±0.04 | 0.233±0.043 | 14.66±1.97 | 3.45±0.07 | 6.26±0.09 | 0.537±0.087 | 9.06±2.20 | 277.76±16.00 | 28.11±1.71 |

Table S6 Physicochemical properties of the soil samples

| Soil sample | pH | Water content  (%) | Organic matter  (g/kg) | Ammonium nitrogen  (mg/kg) | Nitrate nitrogen  (mg/kg) | Total phosphate  (g/kg) | Effective phosphate  (mg/kg) | Total sulfur  (mg/kg) | Effective sulfur  (mg/kg) |
| --- | --- | --- | --- | --- | --- | --- | --- | --- | --- |
| HZHv4 | 7.86±0.04 | 0.155±0.009 | 26.67±1.14 | 1.761±0.194 | 11.77±0.45 | 1.364±0.019 | 7.70±0.87 | 538.32±12.99 | 51.88±1.80 |
| HZBn5 | 8.36±0.04 | 0.179±0.016 | 35.63±0.18 | 5.597±0.263 | 17.60±0.21 | 1.098±0.052 | 25.02±0.25 | 763.83±11.28 | 29.84±1.30 |
| HZTa5 | 8.19±0.04 | 0.189±0.003 | 30.64±0.71 | 4.057±0.139 | 9.06±0.45 | 1.044±0.052 | 53.06±1.30 | 464.71±12.08 | 23.46±1.43 |
| HZHv5 | 7.81±0.02 | 0.198±0.004 | 23.16±0.84 | 3.583±0.099 | 9.99±0.53 | 1.046±0.034 | 11.87±0.59 | 594.33±13.36 | 77.18±2.39 |
| GHBn1 | 8.57±0.03 | 0.152±0.002 | 28.18±2.45 | 4.067±0.050 | 0.65±0.27 | 1.341±0.023 | 27.86±0.47 | 316.07±8.34 | 49.22±2.62 |
| GHTa1 | 8.68±0.03 | 0.080±0.005 | 12.57±1.24 | 4.875±0.562 | 4.69±0.34 | 1.256±0.070 | 5.42±0.65 | 272.04±18.40 | 44.28±1.61 |
| GHHv1 | 8.95±0.03 | 0.062±0.005 | 18.98±2.25 | 2.759±0.319 | 0.24±0.07 | 0.716±0.014 | 13.18±0.29 | 81.82±20.20 | 25.04±0.98 |
| GHBn2 | 8.10±0.10 | 0.175±0.008 | 24.89±0.82 | 4.355±0.142 | 1.76±0.24 | 1.047±0.019 | 0.67±0.19 | 323.10±32.36 | 29.60±0.70 |
| GHTa2 | 8.30±0.01 | 0.147±0.010 | 26.21±1.92 | 3.378±0.308 | 6.77±0.10 | 0.986±0.048 | 12.88±0.57 | 305.70±17.53 | 39.11±0.77 |
| GHHv2 | 8.80±0.16 | 0.164±0.002 | 11.60±1.00 | 4.901±0.405 | 4.12±0.69 | 0.957±0.037 | 2.51±0.49 | 251.84±7.62 | 40.68±1.47 |
| GHBn3 | 8.71±0.06 | 0.046±0.007 | 2.17±0.47 | 2.627±0.257 | 1.18±0.30 | 0.570±0.030 | 6.43±0.98 | 227.14±21.28 | 29.48±2.70 |
| GHTa3 | 8.18±0.07 | 0.106±0.002 | 22.97±1.54 | 3.990±0.244 | 5.32±0.98 | 1.065±0.065 | 9.86±0.90 | 383.75±11.39 | 52.66±3.15 |
| GHHv3 | 8.60±0.07 | 0.038±0.007 | 11.77±0.63 | 2.515±0.324 | 11.78±0.90 | 0.669±0.010 | 14.95±3.91 | 247.87±10.36 | 44.96±3.52 |
| DTBn1 | 8.04±0.06 | 0.175±0.004 | 25.78±0.28 | 4.283±0.139 | 3.57±0.45 | 1.225±0.038 | 47.81±0.85 | 569.52±24.37 | 82.58±1.00 |
| DTTa1 | 7.87±0.15 | 0.173±0.004 | 24.58±1.96 | 1.805±0.245 | 2.50±1.16 | 0.932±0.029 | 21.80±0.54 | 614.29±8.27 | 74.04±8.11 |
| DTHv1 | 8.32±0.25 | 0.181±0.002 | 20.03±1.07 | 3.715±0.152 | 2.89±0.68 | 0.733±0.062 | 1.83±0.38 | 670.67±34.71 | 63.01±3.94 |
| DTHv-Ta1 | 7.63±0.20 | 0.192±0.004 | 39.46±1.43 | 3.223±0.126 | 9.20±0.28 | 1.068±0.043 | 59.10±1.40 | 777.12±22.91 | 51.14±4.81 |

Table S7 Correlation analysis between autotrophic bacterial diversity and soil physicochemical properties

| Soil physicochemical properties | Ace | Chao1 | Shannon | Simpson |
| --- | --- | --- | --- | --- |
| Altitude | –0.023 | –0.044 | 0.105 | 0.180 |
| pH | –0.296 | –0.294 | –0.173 | –0.093 |
| Water content | 0.242 | 0.255 | 0.071 | 0.006 |
| Organic matter | 0.226 | 0.237 | 0.096 | 0.072 |
| Ammonium nitrogen | –0.066 | –0.069 | 0.128 | 0.151 |
| Nitrate nitrogen | 0.165 | 0.161 | 0.027 | –0.023 |
| Total phosphate | 0.035 | 0.046 | 0.018 | 0.061 |
| Effective phosphate | 0.247 | 0.252 | 0.169 | 0.124 |
| Total sulfur | 0.079 | 0.088 | 0.024 | 0.006 |
| Effective sulfur | 0.115 | 0.137 | 0.149 | 0.121 |
| RubisCO activity | 0.073 | 0.089 | 0.257 | 0.300 |

* − correlation is significant at the 0.05 level, ** − correlation is significant at the 0.01 level.
